# LPA_1_ antagonist BMS-986020 changes collagen dynamics and exerts antifibrotic effects in vitro and in patients with idiopathic pulmonary fibrosis

**DOI:** 10.1186/s12931-022-01980-4

**Published:** 2022-03-18

**Authors:** Benjamin E. Decato, Diana Julie Leeming, Jannie Marie Bülow Sand, Aryeh Fischer, Shuyan Du, Scott M. Palmer, Morten Karsdal, Yi Luo, Anne Minnich

**Affiliations:** 1 Research & Early Development, Bristol Myers Squibb, 3401 Princeton Pike, Princeton, NJ 08648 USA; 2 Nordic Bioscience, Herlev Hovedgade 205-207, 2730 Herlev, Denmark; 3 Duke University Medical Center, 2085 Msrb2 2 Genome Ct., Durham, NC 27710 USA

**Keywords:** LPA_1_ antagonist, Collagen, Idiopathic pulmonary fibrosis, Fibrosis, Extracellular matrix, Biomarkers, Scar-in-a-Jar

## Abstract

**Background:**

Idiopathic pulmonary fibrosis (IPF) is a debilitating lung disease with limited treatment options. A phase 2 trial (NCT01766817) showed that twice-daily treatment with BMS-986020, a lysophosphatidic acid receptor 1 (LPA_1_) antagonist, significantly decreased the slope of forced vital capacity (FVC) decline over 26 weeks compared with placebo in patients with IPF. This analysis aimed to better understand the impact of LPA_1_ antagonism on extracellular matrix (ECM)-neoepitope biomarkers and lung function through a post hoc analysis of the phase 2 study, along with an in vitro fibrogenesis model.

**Methods:**

Serum levels of nine ECM-neoepitope biomarkers were measured in patients with IPF. The association of biomarkers with baseline and change from baseline FVC and quantitative lung fibrosis as measured with high-resolution computed tomography, and differences between treatment arms using linear mixed models, were assessed. The Scar-in-a-Jar in vitro fibrogenesis model was used to further elucidate the antifibrotic mechanism of BMS-986020.

**Results:**

In 140 patients with IPF, baseline ECM-neoepitope biomarker levels did not predict FVC progression but was significantly correlated with baseline FVC and lung fibrosis measurements. Most serum ECM-neoepitope biomarker levels were significantly reduced following BMS-986020 treatment compared with placebo, and several of the reductions correlated with FVC and/or lung fibrosis improvement. In the Scar-in-a-Jar in vitro model, BMS-986020 potently inhibited LPA_1_-induced fibrogenesis.

**Conclusions:**

BMS-986020 reduced serum ECM-neoepitope biomarkers, which were previously associated with IPF prognosis. In vitro, LPA promoted fibrogenesis, which was LPA_1_ dependent and inhibited by BMS-986020. Together these data elucidate a novel antifibrotic mechanism of action for pharmacological LPA_1_ blockade.

*Trial registration* ClinicalTrials.gov identifier: NCT01766817; First posted: January 11, 2013; https://clinicaltrials.gov/ct2/show/NCT01766817.

**Supplementary Information:**

The online version contains supplementary material available at 10.1186/s12931-022-01980-4.

## Background

Idiopathic pulmonary fibrosis (IPF) is a progressive and fatal disease characterized by lung fibrosis leading to loss of lung function [1]. IPF is characterized by epithelial damage and changes to the extracellular matrix (ECM) composition, resulting in fibroblast activation and migration into the interstitium, collagen accumulation, and stiffening of the lung tissue [2, 3]. Types I and III collagen are the main structural proteins in the interstitial matrix and are greatly remodeled during pulmonary fibrosis [3]. Type IV collagen is the main constituent of the basement membrane underlying epithelial and endothelial cells lining the airways and vessels [3]. Type VI collagen is located in the interface between the interstitial matrix and the basement membrane; its expression is higher in lungs with IPF compared with healthy lungs [4, 5].

Assessing the ECM turnover provides information about tissue equilibrium, which is the balance between fibrogenesis and fibrolysis. Neoepitope biomarkers can be used to assess ECM remodeling in fibrotic conditions such as IPF [6, 7]; these neoepitope biomarkers measure newly formed epitopes that may be generated by removal of collagen propeptides (reflecting protein formation) or by specific protease-mediated cleavage of mature proteins (reflecting protein degradation). Multiple longitudinal studies have demonstrated that elevated serum levels of these biomarkers are associated with poor prognosis in IPF [8–10].

Although the two antifibrotic treatments for IPF, pirfenidone and nintedanib, can slow forced vital capacity (FVC) decline, these therapies are associated with tolerability considerations [2, 11]. To address this unmet need, blockade of the lysophosphatidic acid (LPA)–LPA receptor 1 (LPA_1_) pathway is currently being studied in clinical trials. LPA promotes normal wound healing and collagen deposition, including fibroblast activation, proliferation, and migration [12]; however, increased LPA levels and activation of LPA_1_ can promote fibrosis and are implicated in IPF pathogenesis [12, 13]. Prior work suggests that LPA_1_ antagonism may be directly antifibrotic, particularly in lung fibrosis [14]. BMS-986020, a first- generation orally bioavailable LPA_1_ antagonist, demonstrated proof-of-mechanism in a phase 2 clinical trial in patients with IPF [15]. Overall, BMS-986020 compared with placebo for 26 weeks slowed FVC decline, with significant differences following 600 mg twice daily (BID) administration. Although BMS-986020 was generally well tolerated in most patients, the clinical program was terminated due to hepatobiliary effects leading to cholecystectomy in three patients. Follow-up in vivo and in vitro analyses determined that the observed hepatobiliary toxicity was specific to BMS-986020 and unrelated to LPA_1_ antagonism [16]. A second- generation LPA_1_ antagonist, BMS-986278, has not shown evidence of hepatobiliary toxicity in nonclinical evaluations or phase 1 studies and is currently in phase 2 development in patients with IPF and progressive fibrotic interstitial lung disease (NCT04308681) [16].

In this study, in vivo and in vitro analyses were performed to enhance our understanding of the impact of LPA_1_ antagonism with BMS-986020 on ECM remodeling and lung function in patients with IPF. The effects of BMS-986020 on nine ECM-neoepitope serum biomarkers were assessed in a post hoc analysis of the phase 2 trial NCT01766817 [15], and the direct effects of BMS-986020 on fibrogenesis were further evaluated in an in vitro Scar-in-a-Jar system. The results from these analyses are presented below.

## Methods

### Collagen neoepitope measurements

Serum ECM-neoepitope biomarkers were measured in 140 patients (placebo: n = 44; 600 mg BMS-986020 once daily (QD): n = 48; 600 mg BMS-986020 BID: n = 48) from a phase 2 trial of BMS-986020 in patients with IPF [15]. The results presented here are restricted to this post hoc analysis of data from the previously described phase 2 study [15]. A subset of patients completed Week 26 of the study, and the patient numbers varied for each biomarker based on sample availability.

Table 1 lists the ECM-neoepitope biomarkers measured. Validated enzyme-linked immunoassays (Nordic Bioscience, Herlev, Denmark) employing neoepitope-specific monoclonal antibodies were used to measure the biomarker levels in serum samples from patients with IPF and/or cell supernatants from the Scar-in-a-Jar assay as previously published [17–28] and below. Serum measurements were performed in duplicates and supernatants in single determinations, all in blinded fashion.

**Table 1:** Biomarker specifications

Biomarker	Description	Process measured
$\mathrm{\alpha }$-SMA [17]	N-terminal of alpha-smooth muscle actin	Myofibroblast marker
FBN-C [18]	C-terminal of fibronectin	Fibronectin formation
C1M [19]	Neoepitope of MMP-2,9,13–mediated degradation of type I collagen	Type I collagen degradation
C3A [20]	Neoepitope of ADAMTS-1,4,8–mediated degradation of type III collagen	Type III collagen degradation
C3M [21]	Neoepitope of MMP-9–mediated degradation of type III collagen	Type III collagen degradation
C4M2 [22]	Neoepitope of MMP-2,9,12–mediated degradation of type IV collagen	Type IV collagen degradation
C6M [23]	Neoepitope of MMP-2–mediated degradation of type VI collagen	Type VI collagen degradation
PRO-C1 [24]	Internal epitope in the N-terminal propeptide of type I collagen	Type I collagen formation
PRO-C3 [25]	Released N-terminal propeptide of type III collagen	Type III collagen formation
PRO-C4 [26]	Internal epitope in the 7S domain of type IV collagen	Type IV collagen formation
PRO-C6 [27]	Released C5 domain of type VI collagen (endotrophin)	Type VI collagen formation
VICM [28]	Neoepitope of MMP-2,8–mediated degradation of citrullinated vimentin	Macrophage marker, inflammation

*ADAMTS* a disintegrin and metalloproteinase with thrombospondin motif; *MMP* matrix metalloproteinase

### Lung function assessments

FVC and quantitative lung fibrosis (QLF) were assessed during the phase 2 study NCT01766817 as published [15]. The QLF scores, which were estimations of the extent of reticular patterns based on high-resolution computed tomography scans, were calculated using a machine learning technique and automated using FIVE steps: (1) denoise; (2) grid-sampling; (3) calculation of selected important texture features; (4) classification with support vector machine; and (5) production of a ratio of the classified fibrotic reticulation to the total grid sample in a percent scale [29–31]. The following formula was used: QLF = counts of classified pulmonary fibrosis/total counts of grid sample [29]. Here, QLF is reported in the whole lung as well as left and right lower lungs of patients in NCT01766817.

### Scar-in-a-Jar experimental design

**Fig. 1:**
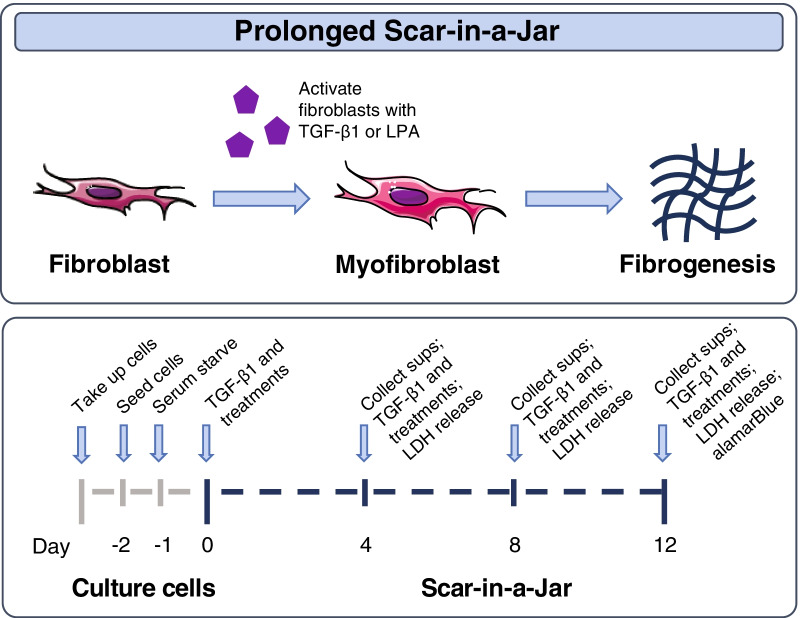
Diagram of Scar-in-a-Jar experimental system. Modified from Rønnow, SR, et al. Respir Res. 2020;21(1):108. Creative Commons Attribution 4.0 International License (https://creativecommons.org/licenses/by/4.0/). *LDH* lactate dehydrogenase; *LPA* lysophosphatidic acid; *Sups* supernatants; *TGF-β1* transforming growth factor-beta 1

Scar-in-a-Jar is an in vitro 3D model of fibrogenesis that uses macromolecular crowding to enhance collagen production and promote collagen crosslinking in cultured fibroblasts (Fig. 1) [32, 33]. In this study, a prolonged system was used to more directly investigate the effects of BMS-986020 on ECM-neoepitope biomarkers. In brief, human lung fibroblasts (cat. no. CC-2512, Lonza, Basel, Switzerland) were cultured in 48-well plates in Dulbecco’s Modified Eagle Medium (DMEM) + GlutaMax with 0.4% fetal bovine serum, 37.5 mg/mL Ficoll 70, 25 mg/mL Ficoll 400, and 1% ascorbic acid. Cells were stimulated with 1 ng/mL transforming growth factor beta 1 (TGF-β1) (cat. no. 100-B-010, R&D Systems, Minneapolis, MN, USA) or 20 µM LPA (cat. no. 360130P, Avanti Polar Lipids, Alabaster, AL, USA) with or without BMS-986020 (0.01, 0.05, 0.1, 0.5, 1, or 5 µM) diluted in dimethyl sulfoxide (DMSO), or vehicle (0.05% DMSO) in four replicates. Cells were cultured at 37 °C with 95% O_2_ and 5% CO_2_ for 12 days, and medium was changed at Day 4 and 8. Supernatants were stored at − 20 °C until biomarker measurements. alamarBlue (cat. no. DAL1100, Invitrogen, Carlsbad, CA, USA) was used to quantify cellular metabolism at Day 0 (prior to drug treatment) and Day 12. Release of lactate dehydrogenase (LDH) was quantified at Day 4, 8, and 12 using the Cytotoxicity Detection KitPLUS (LDH) (cat. no. 04744934001, Roche, Basel, Switzerland).

### Statistical analysis

ECM-neoepitope biomarker association with QLF and FVC was computed using linear regression. Pairwise Spearman and Pearson correlations were computed using the Hmisc R package, and *P* values were adjusted to limit the false discovery rate at α = 0.05 [34, 35]. Linear mixed models were fit to predict log-transformed ECM-neoepitope levels from treatment and time, adjusting for age, sex, and baseline levels, including a random intercept for each patient. The model included an interaction between treatment and time, allowing the slopes for the predicted ECM-neoepitope biomarker levels to differ over time across the different treatments. A likelihood ratio test was performed with and without the interaction term between treatment and time to determine whether this interaction term led to significantly better prediction. A Kruskal–Wallis one-way analysis of variance was used to identify differences between ECM-neoepitope levels at baseline between study groups. No imputation of missing data was performed.

Scar-in-a-Jar data were plotted as mean ± standard error of the mean of four technical replicates. Kruskal–Wallis tests with Dunnett’s multiple comparisons test compared vehicle with BMS-986020 or stimulation (TGF-β1/LPA) alone.

## Results

### QLF and FVC were inversely related in patients with IPF

**Fig. 2:**
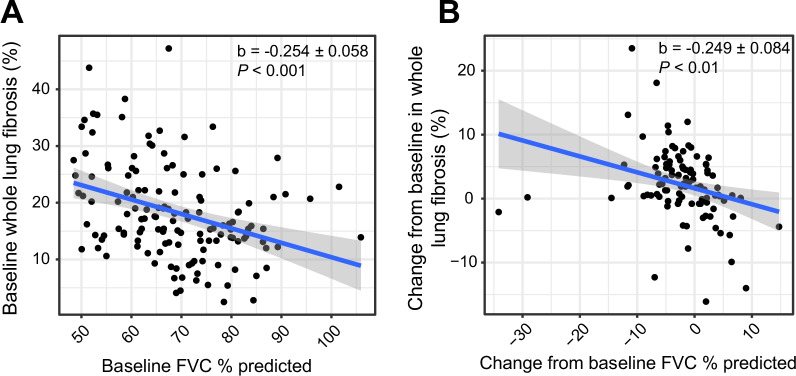
Lung fibrosis (as measured by QLF) correlations with FVC. Scatterplots and linear regression line predicting baseline (**A**) and CFB (**B**) whole lung percent fibrosis from baseline and CFB FVC, respectively. *CFB* change from baseline; *ECM* extracellular matrix; *FVC* forced vital capacity; *QLF* quantitative lung fibrosis

As previously published, the study population included 143 patients with IPF between 40 and 90 years of age [15]. The majority of patients were male (102/143 patients, 71%), the median (range) age was 69 (45–87), and the median (range) FVC and diffusing capacity of the lung for carbon monoxide (DL_CO_) percent predicted were 68 (48–106) and 41 (11–97), respectively. In the current post hoc analysis of 140 patients, baseline QLF and FVC showed inverse relationships, with lower baseline FVC significantly correlating with higher baseline QLF (Fig. 2A, linear regression, b =  − 0.254 ± 0.058, *P* < 0.001). QLF and FVC change over time also showed similar inverse relationships, with FVC decline over the course of the study significantly associating with increases in whole lung QLF (Fig. 2B, b =  − 0.249 ± 0.084, *P* < 0.01).

### ECM-neoepitope biomarker levels were correlated with QLF and FVC in patients with IPF

**Fig. 3:**
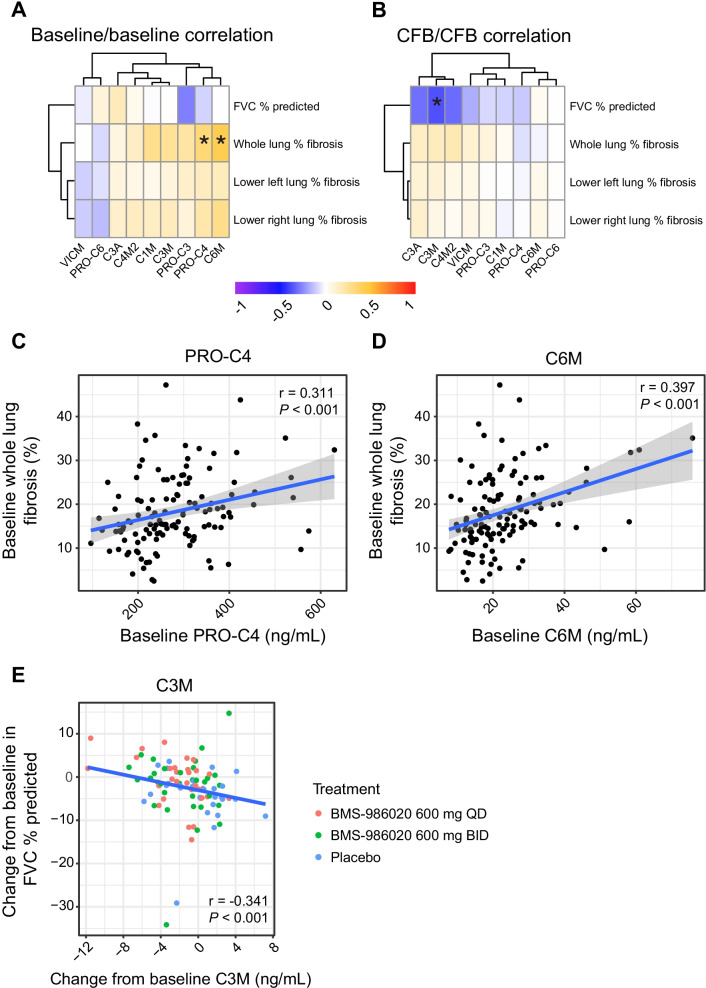
Heatmaps (**A**, **B**) and scatterplots and linear regression of ECM-neoepitope biomarker levels and pulmonary measures (**C**–**E**). **A** Heatmap of pairwise Spearman correlation of baseline ECM-neoepitope biomarker levels with baseline FVC and fibrosis measurements. **B** Heatmap of pairwise Spearman correlation of Week 26 ECM-neoepitope biomarker CFB with FVC and fibrosis CFB. Scatterplots and linear regression of baseline PRO-C4 and C6M levels by baseline whole lung QLF (**C**, **D**) and Week 26 CFB in C3M by Week 26 CFB in FVC, colored by treatment arm (**E**). *P < 0.05. *CFB* change from baseline; *BID* twice daily; *FVC* forced vital capacity; *QD* once daily; *QLF* quantitative lung fibrosis. ECM-neoepitope biomarker abbreviations are defined in Table 1

Baseline ECM-neoepitope biomarker values stratified by treatment are listed in Table 2. At baseline, whole lung QLF was significantly correlated with PRO-C4 (r = 0.311, *P* < 0.001) and C6M (r = 0.397, *P* < 0.001) (Fig. 3A, C, D). While not every correlation was statistically significant after multiple testing correction, a broad trend of positive association between baseline ECM-neoepitope biomarkers and baseline fibrosis in all lung areas was observed.

**Table 2:** Baseline ECM-neoepitope biomarker concentrations stratified by treatment group

ECM-neoepitope biomarker^a^	Baseline biomarker concentration, mean (SEM), ng/mL		
	BMS-986020 600 mg QD	BMS-986020 600 mg BID	Placebo
C1M	36.4 (5.0)	27.3 (2.4)	25.6 (1.7)
C3A	53.3 (2.0)	48.4 (2.7)	43.1 (3.1)
C3M	13.0 (0.6)	11.9 (0.5)	11.5 (0.5)
C4M2*	31.3 (3.6)	24.2 (2.0)	22.4 (1.7)
C6M	25.4 (2.3)	22.5 (1.4)	20.5 (1.2)
PRO-C3	13.7 (1.6)	14.4 (1.2)	13.3 (1.1)
PRO-C4	296.9 (18.3)	282.3 (14.1)	248.5 (10.9)
PRO-C6	10.8 (0.7)	12.6 (0.9)	12.1 (1.0)
VICM	5.4 (0.9)	6.7 (1.6)	5.1 (0.7)

No other neoepitope markers showed significant differences at baseline by treatment arm. ECM-neoepitope biomarker abbreviations are defined in Table 1

*BID* twice daily; *ECM* extracellular matrix; *QD* once daily; *SEM* standard error of the mean

*Significant difference at baseline between treatment arms identified by Kruskal–Wallis test (*P* = 0.045)

**Fig. 4:**
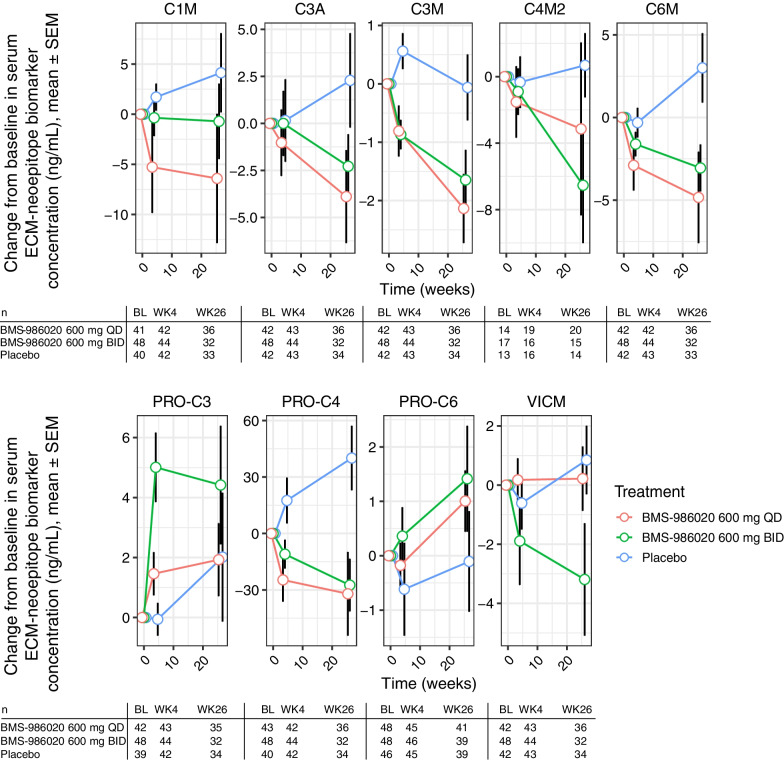
ECM-neoepitope biomarker CFB measurements in patients with IPF from the phase 2 trial NCT01766817. Patient numbers for each ECM-neoepitope biomarker stratified by treatment group and time point are indicated. *BID* twice daily; *BL* baseline; *CFB* change from baseline; *ECM* extracellular matrix; *IPF* idiopathic pulmonary fibrosis; *QD* once daily; *SEM* standard error of the mean; *WK* week. ECM-neoepitope biomarker abbreviations are defined in Table 1

^a^Patient numbers for each ECM-neoepitope biomarker and treatment group are listed in Fig. 4

Change from baseline (CFB) in C3M was negatively correlated with changes in FVC at Week 26 (r =  − 0.341, *P* < 0.001) (Fig. 3B, E). Broad correlations with increases in fibrosis corresponding to increasing levels of ECM-neoepitope biomarkers and decrease in lung function were observed. Investigation of the percent CFB in C6M and QLF in the BMS-986020–treated arms also revealed a significant correlation between increasing C6M levels and increasing QLF (Additional file 1: Table S1). Sample sizes, Spearman correlations, and unadjusted *P* values for baseline/baseline and CFB/CFB correlation analyses are listed in Additional file 1: Table S1.

When stratified by positive versus negative change in fibrosis and FVC, baseline ECM-neoepitope levels largely overlapped, indicating a limited predictive power on disease progression over 26 weeks as measured by FVC in this study (Additional file 1: Fig. S1).

### BMS-986020 modulated ECM-neoepitope biomarker levels

With the relationship between FVC and lung fibrosis established, and the extent to which ECM-neoepitope biomarkers correlated with pulmonary function and fibrosis characterized, the effect of BMS-986020 on serum ECM-neoepitope biomarkers over time was analyzed. Compared with placebo at Week 26, BMS-986020 treatment significantly reduced serum levels of all ECM-neoepitope biomarkers, with the exceptions of PRO-C3 and PRO-C6, in linear mixed model analysis (Fig. 4 and Additional file 1: Fig. S2). The CFB values in ECM-neoepitope biomarker levels at Week 26 are shown in Additional file 1: Table S2, and the likelihood ratio test *P* values and model summaries are shown in Additional file 1: Table S3.

Given the known relationship between PRO-C3 and PRO-C6 with liver dysfunction [36], it was possible that their unexpected increases were associated with the hepatobiliary toxicity observed in the BMS-986020–treated population. In the BMS-986020 treatment arms, the Week 26 CFB in PRO-C3 was significantly correlated with changes in direct bilirubin and aspartate aminotransferase (AST) levels. Week 26 CFB in PRO-C6 was also correlated with direct bilirubin level (Additional file 1: Fig. S3). In contrast, collagen degradation biomarkers did not positively correlate with changes in liver enzyme levels.

### BMS-986020 inhibited LPA_1_-induced fibrogenesis in the Scar-in-a-Jar in vitro model

**Fig. 5:**
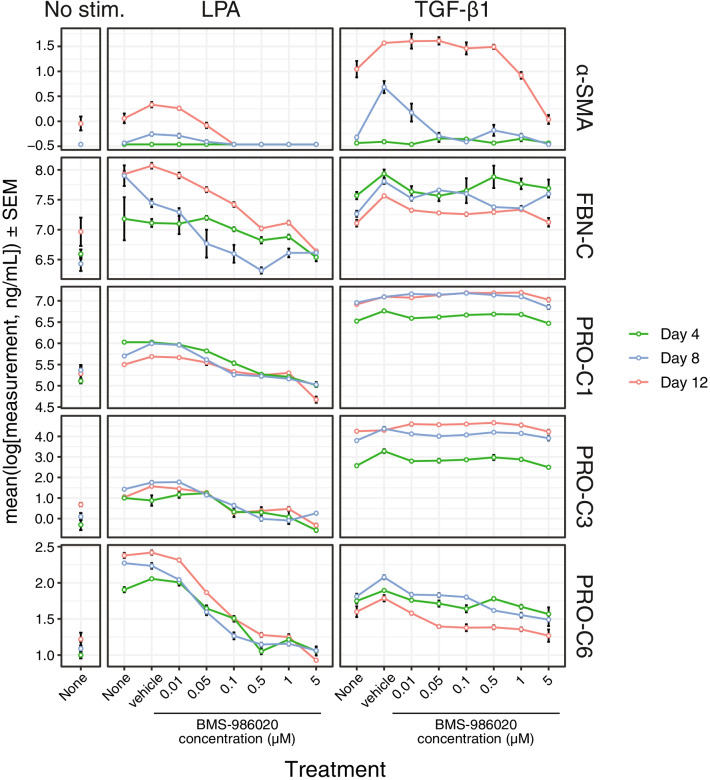
Effects of BMS-986020 on LPA- or TGF-β1–stimulated fibrogenesis in the Scar-in-a-Jar in vitro model. Shown are ECM-neoepitope biomarkers over time for untreated, vehicle-, and BMS-986020–treated fibroblasts. Similar results were obtained in a prior experiment. *ECM* extracellular matrix; *LPA* lysophosphatidic acid; *SEM* standard error of the mean; *Stim* stimulation; *TGF-β1* transforming growth factor-beta 1. ECM-neoepitope biomarker abbreviations are defined in Table 1

For direct assessment of the effects of LPA and LPA_1_ antagonism on ECM-neoepitope biomarkers, the prolonged Scar-in-a-Jar in vitro fibrogenesis model was used. These Scar-in-a-Jar studies evaluated biomarkers of collagen formation (PRO-C1, PRO-C3, and PRO-C6), α-SMA, and FBN-C but could not analyze ECM degradation biomarkers due to the lack of collagen-degrading proteases in the system. Compared with no stimulation, LPA increased the levels of $\mathrm{\alpha }$-SMA, FBN-C, PRO-C1, PRO-C3, and PRO-C6 in culture supernatant (Fig. 5, note log scale). LPA induced higher levels of FBN-C (Days 8 and 12) and PRO-C6 (all days) and lower levels of α-SMA, PRO-C1, and PRO-C3 (all days) than did TGF-β1. For each biomarker, stimulation, and timepoint trio, Kruskal–Wallis test followed by *P* value false discovery rate adjustment revealed significant differences in the means across 27 of the 30 combinations (Additional file 1: Table S4). Dunnett’s test comparing each BMS-986020 concentration (as well as untreated) to vehicle at Day 4, 8, and 12 revealed significant dose-dependent reductions in the five biomarkers measured in LPA-stimulated cells, with smaller but significant effects on $\mathrm{\alpha }$-SMA and PRO-C6 in the TGF-β1–stimulated condition (Additional file 1: Table S5). In the LPA-stimulated condition, BMS-986020-mediated inhibition on production of all biomarkers appeared maximal at approximately 0.5 μM (Fig. 5).

BMS-986020–mediated decreases in metabolic activity, as measured by alamarBlue, were observed in the LPA-stimulated (*P* < 0.01) and unstimulated (*P* < 0.05) cells on Day 12 (Additional file 1: Fig. S4 and Table S6A); however, these effects occurred at higher concentrations and were of lower magnitude than the effects on biomarker production (Additional file 1: Fig. S5). Unstimulated cells treated with BMS-986020 also had significantly slower increases in ECM-neoepitope biomarker levels over time (Additional file 1: Fig. S6 and Table S6B). Other experiments showed that BMS-986020 had no effect on LDH release at up to 10 μM, indicating lack of cytotoxicity.

## Discussion

A previous report demonstrated that BMS-986020, a first-generation LPA_1_ antagonist, significantly slowed FVC decline compared with placebo in a phase 2 clinical trial in patients with IPF [15]. The current analysis reports on a subset of the ECM-neoepitope biomarkers that have a well-established association with IPF disease progression for possible response prediction and disease/treatment monitoring.

Median survival for IPF is 3–5 years post-diagnosis [2, 37], but prognosis can be variable and unpredictable. Numerous published studies have aimed to better predict prognosis using circulating biomarkers [38], but this study focused on ECM-neoepitope biomarkers due to the hypothesized antifibrotic mechanism of action of LPA_1_ inhibition. The prognostic power of these biomarkers for IPF has been reported in the large, multicenter, and longitudinal PROFILE [9] and INMARK [39, 40] studies. In the PROFILE study (n = 140, baseline mean [standard deviation, SD] percent predicted FVC 79.8% [20.4%]), VICM, and ECM-degradation biomarkers C1M, C3A, C3M, and C6M were associated with IPF disease progression [8, 9]. High baseline serum concentrations of collagen formation biomarkers, PRO-C3 and PRO-C6, were also associated with more rapid disease progression (≥ 10% FVC decline or death) [9]. The latter findings were consistent with results from the PFBIO cohort, a real-world cohort of Danish patients with mild IPF (n = 185, baseline mean [SD] percent predicted FVC 89.7% [19.3]) [10].

In addition to the strong implication of these biomarkers in IPF disease prognosis as measured by survival or composite endpoints, other studies have recently assessed their association with lung function decline alone. In the PFBIO cohort, high baseline serum levels of C1M and PRO-C3 were associated with larger lung function declines over 1 year as measured by FVC and DL_CO_ [10, 41]. An association between measurable clinical outcomes such as lung function decline is an important indicator of the potential utility of these biomarkers for monitoring therapeutic response or disease progression.

Baseline serum levels of ECM-neoepitope biomarkers did not predict treatment response with BMS-986020 as measured by FVC or QLF in this study; however, these biomarkers changed over the 6-month study period and responded to BMS-986020 treatment. ECM-neoepitope biomarker levels increased over the 6-month study duration in placebo-treated patients, reflecting disease worsening, but BMS-986020 treatment for 6 months decreased C1M, C3A, C3M, C4M2, C6M, PRO-C4, and VICM levels relative to baseline and placebo. The changes in C3M and PRO-C4 levels were seen after 4 weeks of treatment, suggesting a direct effect of BMS-986020 on collagen turnover. The drug-induced changes for C3M and C6M in this study (Additional file 1: Table S2) may be clinically meaningful as they were similar in magnitude to the 3-month changes observed in stable versus progressive IPF using a composite endpoint in the PROFILE study [8, 9].

Furthermore, both baseline biomarker levels and treatment effects on biomarkers were related to pulmonary outcomes (FVC and QLF) in this study. At baseline, C6M levels were significantly correlated to QLF; at Week 26, CFB in C3M was significantly negatively correlated with FVC changes. Consistent with this, in categorical analyses, patients with no FVC decline tended to have a larger mean decrease in C3M level at Week 26 compared with patients whose FVC declined [42]. Patients with no worsening of fibrosis displayed larger mean decrease in C6M level than did patients whose lung fibrosis increased at Week 26 [42]. Although this study was not powered to detect the correlations, this post hoc analysis was used to look for overall trends in the data to support the hypothesis regarding the mechanism of action of LPA_1_ antagonism in IPF.

In contrast to the effect of drug-induced decreases in the levels of ECM-neoepitopes such as collagen degradation biomarkers, the increases in PRO-C3 and PRO-C6 levels were unexpected; however, PRO-C3 at baseline showed moderate but significant negative correlation with FVC percent predicted (Additional file 1: Table S1). BMS-986020 treatment was associated with increased incidence of hepatic enzyme or bilirubin elevations (≥ 3× upper limit of normal) in 7/48 patients (600 mg QD) and 15/48 (600 mg BID) compared with those who received placebo (0/47) [15]. Retrospective nonclinical investigations indicate that this hepatobiliary toxicity is an off-target effect specific to BMS-986020 and is unlikely to affect structurally distinct LPA_1_ antagonists [16, 43]. Both PRO-C3 and PRO-C6 have been strongly associated with prognosis in other fibrotic diseases; PRO-C3 is a well-established biomarker for nonalcoholic steatohepatitis and other fibrotic liver diseases [44], and PRO-C6 is also elevated in liver fibrosis, but to a lesser extent than is PRO-C3 [36]. Although it can be difficult to attribute changes in levels of circulating biomarkers to a particular organ or disease, BMS-986020–mediated increases in PRO-C3 and PRO-C6 levels in the current clinical study may be ascribed to hepatic effects. Evidence in favor of this hypothesis includes the correlation of these changes with elevated liver enzymes and the in vitro data showing that BMS-986020 decreased PRO-C3 and PRO-C6 production. In patients treated with BMS-986020, Week 26 CFB in PRO-C6 exhibited a significant positive correlation with direct bilirubin, and CFB in PRO-C3 correlated with bilirubin and AST levels. Notably, collagen degradation biomarkers did not positively correlate with changes in liver enzymes. These positive associations between PRO-C3 and PRO-C6 and markers of liver injury could explain their unexpected increases in BMS-986020–treated groups [36].

The Scar-in-a-Jar studies herein demonstrated that LPA stimulates lung-derived fibroblast activation by increasing collagen synthesis in a manner that was completely inhibited by an LPA_1_ antagonist. To our knowledge, this is the first report that LPA exerts direct and LPA_1_-dependent fibrogenic effects. Compared with no stimulation, PRO-C1, PRO-C3, and PRO-C6 production increased following LPA stimulation, although effects on fibrogenesis were not limited to collagen production as levels of α-SMA and FBN-C also increased. BMS-986020 significantly reduced levels of biomarkers of collagen production in response to LPA and markedly inhibited FBN-C production, consistent with a more general effect on fibroblast invasion into the ECM and activation via integrin binding [18]. The approximate IC_100_ for BMS-986020–mediated inhibition of ECM formation (100–500 nM) is consistent with the plasma trough drug levels observed in the 600 mg BID treatment group of the phase 2 IPF study (median 701 nM Week 26, unpublished observations).

Limited data are available regarding the effects of existing or other experimental pulmonary fibrosis therapies on ECM-neoepitope biomarkers. In the INMARK study, nintedanib did not affect C1M or C3M after 12 weeks [39] but transiently increased and decreased PRO-C3 and PRO-C6 levels, respectively, after 4 and 12 weeks of treatment [45]. Neither pirfenidone nor nintedanib appeared to affect PRO-C3 or PRO-C6 levels over 12 months in the PFBIO study, although the study was not powered to detect treatment effects [46]. Rapid effects were seen with omipalisib in a phase 1b study, which reduced PRO-C3 and PRO-C6 levels in patients with IPF within 10 days of treatment [47]. This finding was also consistent with in vitro effects reported in the Scar-in-a-Jar model [33].

In contrast to types I, III, and VI collagens, type IV is a basement membrane collagen [3], and the behavior of its degradation and formation neoepitopes, C4M2 and PRO-C4, has not been previously reported in IPF. In the current study, BMS-986020 treatment significantly reduced PRO-C4 level, which warrants further analysis of PRO-C4 in additional IPF cohorts. The data highlight the potential importance of the combination of interstitial matrix and basement membrane effects of antifibrotic therapy.

Beyond collagen formation and degradation dynamics, another critical aspect of IPF prognosis is *MUC5B* genetic status. The prevalence of a common single nucleotide polymorphism, rs35705950, gain-of-function T-allele is higher in patients with IPF (34–38%) compared with control populations (9–11%) [48, 49] but is paradoxically associated with improved survival [37]. Notably, the *MUC5B* genotype was excluded as a covariate in the models in the current study given the absence of a specific effect of *MUC5B* genotype on FVC response to BMS-986020 [50]. The findings that *MUC5B* status has no interactive effect with treatment response are unsurprising because the mechanism of action of BMS-986020 is not thought to involve *MUC5B*; similarly, the *MUC5B* variant status did not affect treatment with pirfenidone [51].

The present study has multiple limitations, namely small sample size (although this is typical for phase 2 IPF studies) and short duration of 6 months. The latter limitation may not allow for easily measurable effects of therapeutic intervention on FVC and QLF since these measures change slowly over time.

## Conclusions

The data reported herein support the antifibrotic effects of pharmacological LPA_1_ antagonism and extend the scope of these effects to collagen turnover in patients with IPF. Furthermore, the BMS-986020–induced decreases in C3M and C6M levels are associated with more favorable outcomes for FVC and QLF, respectively. ECM-neoepitope biomarkers have potential value to monitor treatment response and disease progression/regression in future pulmonary fibrosis clinical trials of antifibrotic drugs such as BMS-986278, a second- generation LPA_1_ antagonist. The findings support the clinical development of BMS-986278 in a large global phase 2 trial in patients with IPF and progressive fibrotic interstitial lung disease (NCT04308681).

## Supplementary Information

**Additional file 1: Table S1.** Patient numbers, Spearman’s r, and unadjusted *P* values for baseline/baseline, CFB/CFB, %CFB/%CFB (all arms), and %CFB/%CFB (BMS-986020–treated arms) correlations. **Table S2.** Change from baseline in ECM-neoepitope biomarker concentrations at Week 26 stratified by treatment group. **Table S3**. B-H–adjusted *P* values of LRT for treatment*timepoint interactive term significance, and model summaries for all linear mixed models fitted. **Table S4.** Kruskal–Wallis one-way analysis of variance and B-H–adjusted *P* values for each group tested. **Table S5.** Adjusted *P* values from Dunnett’s test of untreated and each BMS-986020 dose with vehicle, for all combinations of fibrosis induction, timepoint, and biomarker in a Scar-in-a-Jar model. **Table S6.** alamarBlue Kruskal–Wallis test raw and adjusted *P* values (A) and likelihood ratio test *P* values testing for significant improvement to model fit when including BMS-986020 treatment status in unstimulated group (B). **Figure S1.** Density plots showing baseline ECM-neoepitope biomarker levels stratified into patient cohorts displaying **A** more or less fibrosis and **B** FVC decline or no FVC decline at Week 26. **Figure S2.** Mean measurement (ng/mL) of ECM-neoepitope biomarkers in patients with IPF from the phase 2 trial NCT01766817. **Figure S3.** Pairwise Spearman and Pearson correlation analyses of Week 26 CFB in clinical laboratory liver biomarkers and ECM-neoepitope biomarkers in patients treated with BMS-986020. **Figure S4.** alamarBlue-measured metabolic activity of LPA- and TGF-β1–stimulated fibroblasts at Day 0 and Day 12, stratified by treatment. **Figure S5.** Fold change between untreated, vehicle-treated, and BMS-986020–treated levels of each ECM-neoepitope biomarker. **Figure S6.** Linear regression of change in ECM-neoepitope biomarker levels over time by treatment in unstimulated Scar-in-a-Jar primary fibroblasts.

## Data Availability

BMS policy on data sharing may be found at https://www.bms.com/researchers-and-partners/independent-research/data-sharing-request-process.html.

## Abbreviations

AST: Aspartate aminotransferase
BID: Twice daily
CFB: Change from baseline
DL_CO_: Diffusing capacity of the lung for carbon monoxide
ECM: Extracellular matrix
FVC: Forced vital capacity
IPF: Idiopathic pulmonary fibrosis
LDH: Lactate dehydrogenase
LPA: Lysophosphatidic acid
LPA_1_: Lysophosphatidic acid receptor 1
QD: Once daily
TGF-β1: Transforming growth factor-beta 1
QLF: Quantitative lung fibrosis
