# Supplementary material for: LPA1 antagonist BMS-986020 changes collagen dynamics and exerts antifibrotic effects in vitro and in patients with idiopathic pulmonary fibrosis
Source: Respir Res. 2022 Mar 18;23:61. doi: 10.1186/s12931-022-01980-4 (PMC8933988; doi:10.1186/s12931-022-01980-4)
Supplement: Supplementary file 1 — Additional file 1: Table S1. Patient numbers, Spearman’s r, and unadjusted P values for baseline/baseline, CFB/CFB, %CFB/%CFB (all arms), and %CFB/%CFB (BMS-986020–treated arms) correlations. Table S2. Change from baseline in ECM-neoepitope biomarker concentrations at Week 26 stratified by treatment group. Table S3. B-H–adjusted P values of LRT for treatment*timepoint interactive term significance, and model summaries for all linear mixed models fitted. Table S4. Kruskal–Wallis one-way analysis of variance and B-H–adjusted P values for each group tested. Table S5. Adjusted P values from Dunnett’s test of untreated and each BMS-986020 dose with vehicle, for all combinations of fibrosis induction, timepoint, and biomarker in a Scar-in-a-Jar model. Table S6. alamarBlue Kruskal–Wallis test raw and adjusted P values (A) and likelihood ratio test P values testing for significant improvement to model fit when including BMS-986020 treatment status in unstimulated group (B). Figure S1. Density plots showing baseline ECM-neoepitope biomarker levels stratified into patient cohorts displaying A more or less fibrosis and B FVC decline or no FVC decline at Week 26. Figure S2. Mean measurement (ng/mL) of ECM-neoepitope biomarkers in patients with IPF from the phase 2 trial NCT01766817. Figure S3. Pairwise Spearman and Pearson correlation analyses of Week 26 CFB in clinical laboratory liver biomarkers and ECM-neoepitope biomarkers in patients treated with BMS-986020. Figure S4. alamarBlue-measured metabolic activity of LPA- and TGF-β1–stimulated fibroblasts at Day 0 and Day 12, stratified by treatment. Figure S5. Fold change between untreated, vehicle-treated, and BMS-986020–treated levels of each ECM-neoepitope biomarker. Figure S6. Linear regression of change in ECM-neoepitope biomarker levels over time by treatment in unstimulated Scar-in-a-Jar primary fibroblasts. [file 12931_2022_1980_MOESM1_ESM.docx]

**ADDITIONAL MATERIALS**

**LPA_1_ antagonist BMS-986020 changes collagen dynamics and exerts antifibrotic effects in vitro and in patients with idiopathic pulmonary fibrosis**

Benjamin E. Decato,^a^ Diana Julie Leeming,^b^ Jannie Marie Bülow Sand,^b^ Aryeh Fischer,^a^ Shuyan Du,^a^ Scott M. Palmer,^c^ Morten Karsdal,^b^ Yi Luo,^a^ Anne Minnich^a^

^a^ Research & Early Development, Bristol Myers Squibb, Princeton, NJ, USA

^b^ Nordic Bioscience, Herlev, Denmark

^c^ Duke University Medical Center, Durham NC, USA

**Table S1.** Patient numbers, Spearman’s r, and unadjusted *P* values for baseline/baseline, CFB/CFB, %CFB/%CFB (all arms), and %CFB/%CFB (BMS-986020–treated arms) correlations^a^

| Baseline/baseline | | | | |
| --- | --- | --- | --- | --- |
| n’s | | | | |
|  | FVC % PREDICTED | Whole lung % fibrosis | Lower left lung % fibrosis | Lower right lung % fibrosis |
| C1M | 128 | 126 | 126 | 126 |
| C3A | 130 | 128 | 128 | 128 |
| C3M | 131 | 129 | 129 | 129 |
| C4M2 | 42 | 42 | 42 | 42 |
| C6M | 131 | 129 | 129 | 129 |
| PRO-C3 | 128 | 126 | 126 | 126 |
| PRO-C4 | 129 | 127 | 127 | 127 |
| PRO-C6 | 140 | 138 | 138 | 138 |
| VICM | 131 | 129 | 129 | 129 |
| Spearman's r | | | | |
|  | FVC % PREDICTED | Whole lung % fibrosis | Lower left lung % fibrosis | Lower right lung % fibrosis |
| C1M | -0.005 | 0.257 | 0.043 | 0.136 |
| C3A | 0.159 | 0.069 | 0.075 | 0.118 |
| C3M | 0.005 | 0.234 | 0.079 | 0.156 |
| C4M2 | 0.037 | 0.130 | 0.055 | 0.141 |
| C6M | -0.001 | 0.397 | 0.152 | 0.266 |
| PRO-C3 | -0.250 | 0.210 | 0.117 | 0.087 |
| PRO-C4 | -0.085 | 0.311 | 0.141 | 0.190 |
| PRO-C6 | 0.083 | -0.072 | -0.059 | -0.131 |
| VICM | -0.035 | -0.002 | -0.090 | -0.099 |
| Unadjusted *P* values | | | | |
|  | FVC % PREDICTED | Whole lung % fibrosis | Lower left lung % fibrosis | Lower right lung % fibrosis |
| C1M | 0.952 | **0.004** | 0.631 | 0.130 |
| C3A | 0.071 | 0.439 | 0.403 | 0.184 |
| C3M | 0.952 | **0.008** | 0.373 | 0.078 |
| C4M2 | 0.817 | 0.411 | 0.729 | 0.374 |
| C6M | 0.991 | **< 0.001** | 0.084 | **0.002** |
| PRO-C3 | **0.004** | **0.018** | 0.192 | 0.331 |
| PRO-C4 | 0.338 | **< 0.001** | 0.114 | **0.033** |
| PRO-C6 | 0.329 | 0.402 | 0.494 | 0.126 |
| VICM | 0.692 | 0.985 | 0.308 | 0.264 |
| CFB/CFB | | | | |
| n’s | | | | |
|  | FVC % PREDICTED | Whole lung % fibrosis | Lower left lung % fibrosis | Lower right lung % fibrosis |
| C1M | 88 | 84 | 84 | 84 |
| C3A | 90 | 86 | 86 | 86 |
| C3M | 91 | 87 | 87 | 87 |
| C4M2 | 17 | 16 | 16 | 16 |
| C6M | 90 | 86 | 86 | 86 |
| PRO-C3 | 87 | 83 | 83 | 83 |
| PRO-C4 | 89 | 85 | 85 | 85 |
| PRO-C6 | 116 | 101 | 101 | 101 |
| VICM | 91 | 87 | 87 | 87 |
| Spearman's r | | | | |
|  | FVC % PREDICTED | Whole lung % fibrosis | Lower left lung % fibrosis | Lower right lung % fibrosis |
| C1M | -0.085 | < 0.001 | 0.026 | -0.021 |
| C3A | -0.280 | 0.096 | 0.131 | 0.114 |
| C3M | -0.341 | 0.091 | 0.152 | 0.059 |
| C4M2 | -0.289 | 0.053 | 0.174 | 0.028 |
| C6M | 0.027 | 0.043 | -0.021 | 0.056 |
| PRO-C3 | -0.075 | 0.010 | 0.073 | 0.038 |
| PRO-C4 | -0.105 | -0.018 | -0.061 | -0.004 |
| PRO-C6 | 0.002 | 0.004 | 0.002 | 0.008 |
| VICM | -0.156 | 0.054 | 0.102 | 0.060 |
| Unadjusted *P* values | | | | |
|  | FVC % PREDICTED | Whole lung % fibrosis | Lower left lung % fibrosis | Lower right lung % fibrosis |
| C1M | 0.434 | 0.999 | 0.813 | 0.848 |
| C3A | **0.008** | 0.379 | 0.228 | 0.296 |
| C3M | **< 0.001** | 0.402 | 0.161 | 0.590 |
| C4M2 | 0.260 | 0.846 | 0.520 | 0.918 |
| C6M | 0.798 | 0.697 | 0.849 | 0.610 |
| PRO-C3 | 0.489 | 0.930 | 0.515 | 0.734 |
| PRO-C4 | 0.325 | 0.872 | 0.580 | 0.968 |
| PRO-C6 | 0.986 | 0.970 | 0.985 | 0.935 |
| VICM | 0.139 | 0.618 | 0.348 | 0.583 |
| %CFB/%CFB, all arms | | | | |
| n’s | | | | |
|  | FVC % PREDICTED | Whole lung % fibrosis | Lower left lung % fibrosis | Lower right lung % fibrosis |
| C1M | 88 | 84 | 84 | 84 |
| C3A | 90 | 86 | 86 | 86 |
| C3M | 91 | 87 | 87 | 87 |
| C4M2 | 17 | 16 | 16 | 16 |
| C6M | 90 | 86 | 86 | 86 |
| PRO-C3 | 87 | 83 | 83 | 83 |
| PRO-C4 | 89 | 85 | 85 | 85 |
| PRO-C6 | 116 | 101 | 101 | 101 |
| VICM | 91 | 87 | 87 | 87 |
| Spearman's r | | | | |
|  | FVC % PREDICTED | Whole lung % fibrosis | Lower left lung % fibrosis | Lower right lung % fibrosis |
| C1M | -0.100 | -0.001 | -0.008 | 0.009 |
| C3A | -0.300 | 0.115 | 0.175 | 0.131 |
| C3M | -0.348 | 0.146 | 0.185 | 0.124 |
| C4M2 | -0.331 | 0.029 | 0.200 | -0.035 |
| C6M | 0.060 | 0.134 | 0.084 | 0.154 |
| PRO-C3 | -0.065 | -0.006 | -0.007 | -0.052 |
| PRO-C4 | -0.039 | -0.005 | 0.001 | 0.021 |
| PRO-C6 | 0.016 | 0.006 | -0.011 | -0.003 |
| VICM | -0.102 | 0.044 | 0.031 | 0.034 |
| Unadjusted *P* values | | | | |
|  | FVC % PREDICTED | Whole lung % fibrosis | Lower left lung % fibrosis | Lower right lung % fibrosis |
| C1M | 0.353 | 0.993 | 0.945 | 0.933 |
| C3A | **0.004** | 0.293 | 0.106 | 0.228 |
| C3M | **< 0.001** | 0.177 | 0.086 | 0.251 |
| C4M2 | 0.195 | 0.914 | 0.458 | 0.897 |
| C6M | 0.576 | 0.218 | 0.439 | 0.157 |
| PRO-C3 | 0.551 | 0.958 | 0.950 | 0.643 |
| PRO-C4 | 0.714 | 0.964 | 0.992 | 0.849 |
| PRO-C6 | 0.868 | 0.954 | 0.911 | 0.973 |
| VICM | 0.338 | 0.688 | 0.775 | 0.754 |
| %CFB/%CFB, (BMS-986020–treated arms) | | | | |
| n’s | | | | |
|  | FVC % PREDICTED | Whole lung % fibrosis | Lower left lung % fibrosis | Lower right lung % fibrosis |
| C1M | 62 | 59 | 59 | 59 |
| C3A | 63 | 60 | 60 | 60 |
| C3M | 63 | 60 | 60 | 60 |
| C4M2 | 13 | 12 | 12 | 12 |
| C6M | 63 | 60 | 60 | 60 |
| PRO-C3 | 61 | 58 | 58 | 58 |
| PRO-C4 | 63 | 60 | 60 | 60 |
| PRO-C6 | 80 | 69 | 69 | 69 |
| VICM | 63 | 60 | 60 | 60 |
| Spearman's r | | | | |
|  | FVC % PREDICTED | Whole lung % fibrosis | Lower left lung % fibrosis | Lower right lung % fibrosis |
| C1M | -0.103 | 0.049 | 0.050 | 0.034 |
| C3A | -0.246 | 0.180 | 0.257 | 0.169 |
| C3M | -0.319 | 0.110 | 0.135 | 0.055 |
| C4M2 | -0.412 | 0.021 | 0.126 | -0.084 |
| C6M | -0.008 | 0.274 | 0.216 | 0.260 |
| PRO-C3 | -0.233 | 0.069 | 0.092 | 0.012 |
| PRO-C4 | -0.053 | 0.024 | 0.034 | 0.024 |
| PRO-C6 | -0.026 | -0.003 | -0.009 | -0.006 |
| VICM | -0.160 | 0.009 | < 0.001 | -0.006 |
| Unadjusted *P* values | | | | |
|  | FVC % PREDICTED | Whole lung % fibrosis | Lower left lung % fibrosis | Lower right lung % fibrosis |
| C1M | 0.424 | 0.713 | 0.708 | 0.797 |
| C3A | 0.052 | 0.168 | **0.048** | 0.196 |
| C3M | **0.011** | 0.403 | 0.305 | 0.676 |
| C4M2 | 0.162 | 0.948 | 0.697 | 0.795 |
| C6M | 0.950 | **0.034** | 0.098 | **0.045** |
| PRO-C3 | 0.070 | 0.609 | 0.493 | 0.931 |
| PRO-C4 | 0.681 | 0.856 | 0.794 | 0.857 |
| PRO-C6 | 0.822 | 0.982 | 0.939 | 0.960 |
| VICM | 0.210 | 0.945 | 0.996 | 0.966 |

^a^ *P* < 0.05 values are shown in bold. CFB, change from baseline; FVC, forced vital capacity.

**Figure S1.** Density plots showing baseline ECM-neoepitope biomarker levels stratified into patient cohorts displaying (A) more or less fibrosis and (B) FVC decline or no FVC decline at Week 26


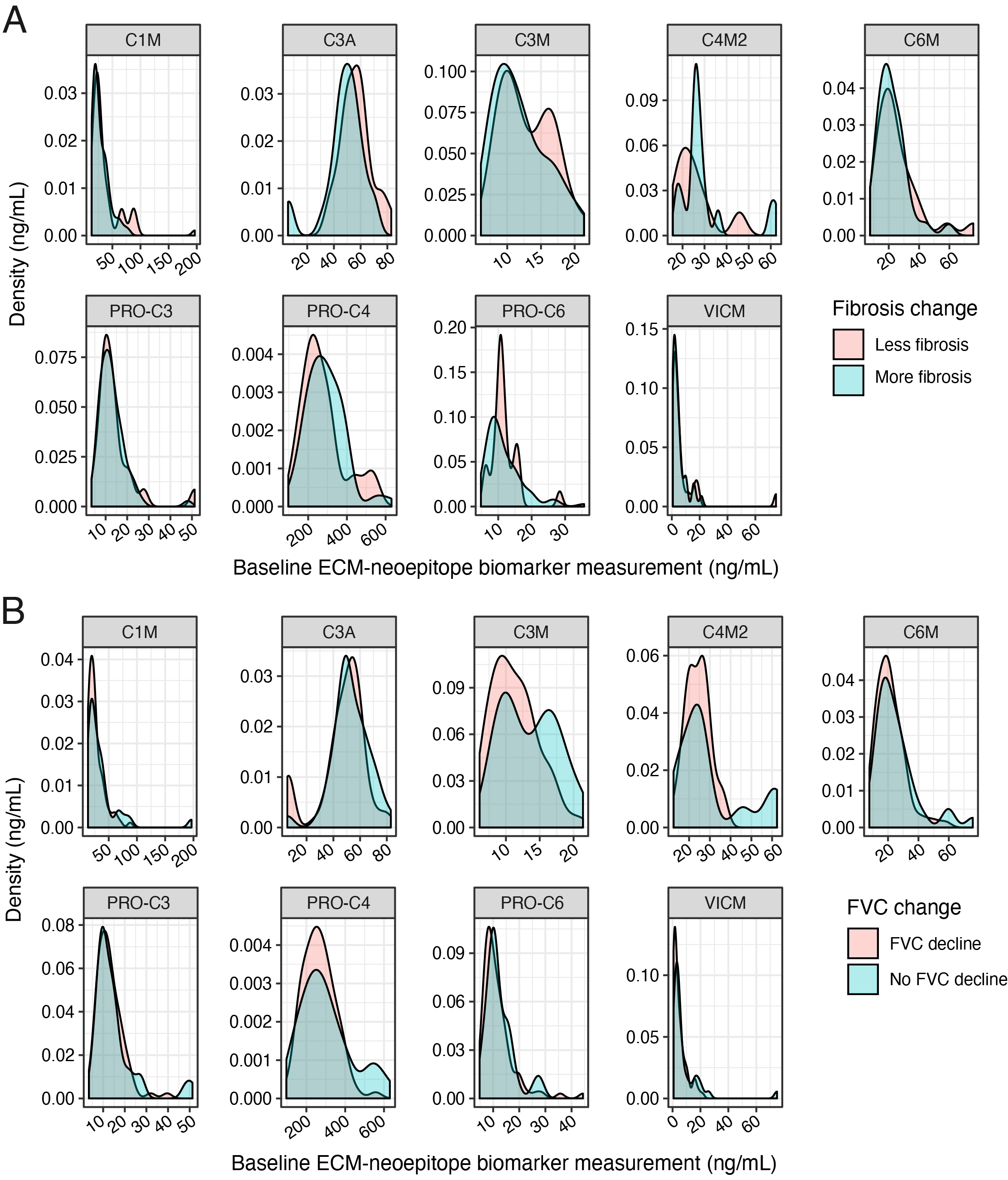


ECM, extracellular matrix; FVC, forced vital capacity.

**Figure S2.** Mean measurement (ng/mL) of ECM-neoepitope biomarkers in patients with IPF from the phase 2 trial NCT01766817


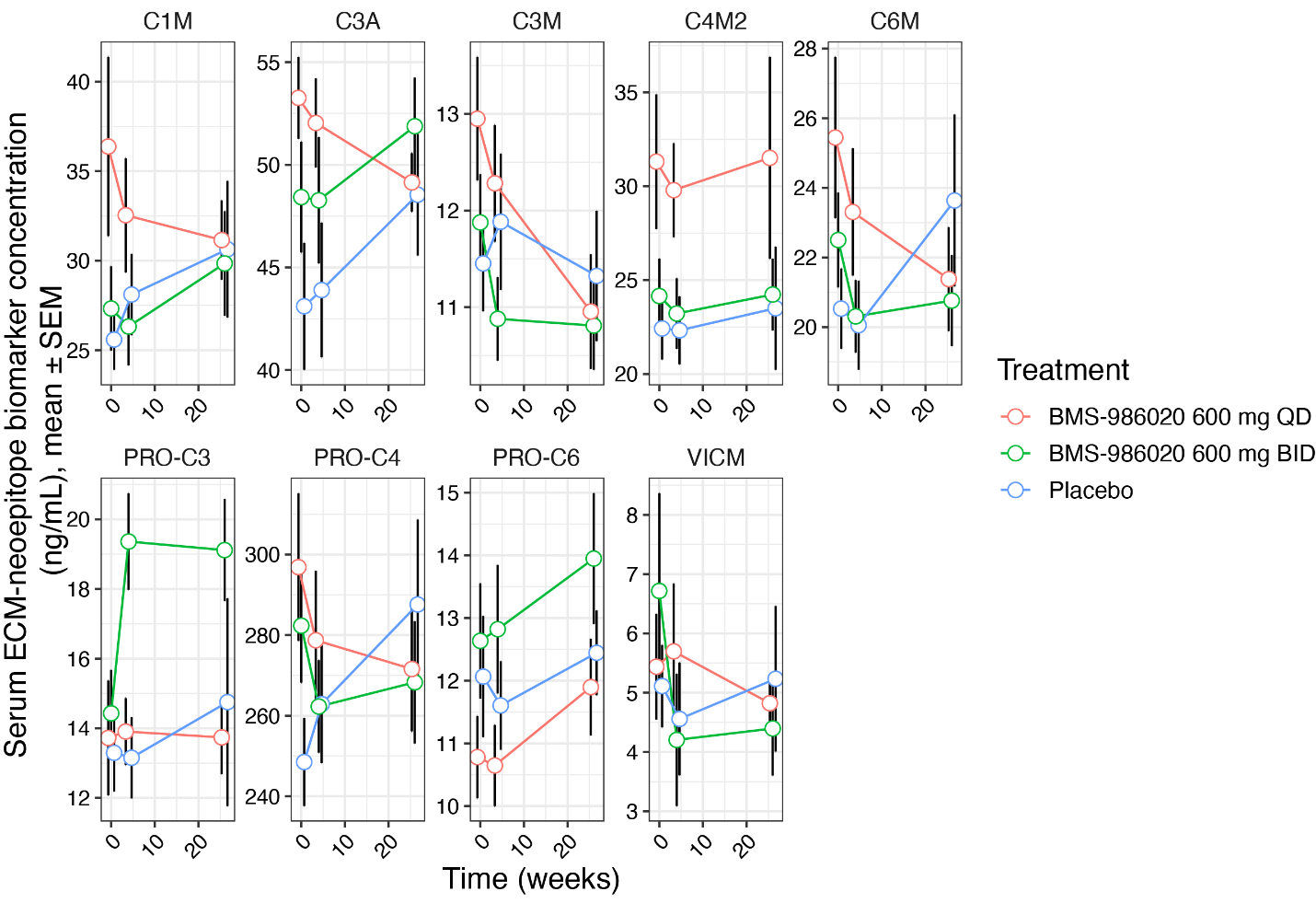


BID, twice daily; ECM, extracellular matrix; IPF, idiopathic pulmonary fibrosis; QD, once daily; SEM, standard error of the mean.

**Table S2.** Change from baseline in ECM-neoepitope biomarker concentrations at Week 26 stratified by treatment group

| Biomarker | Week 26 CFB, mean (SEM), ng/mL | | |
| --- | --- | --- | --- |
|  | BMS-986020  600 mg QD | BMS-986020  600 mg BID | Placebo |
| C1M | -6.4 (6.5) | -0.7 (3.8) | 4.1 (4.0) |
| C3A | -3.9 (2.5) | -2.3 (1.7) | 2.3 (2.5) |
| C3M | -2.1 (0.6) | -1.6 (0.5) | -0.1 (0.6) |
| C4M2 | -3.1 (5.2) | -6.5 (3.5) | 0.7 (1.9) |
| C6M | -4.8 (2.8) | -3.1 (1.4) | 3.0 (2.1) |
| PRO-C3 | 1.9 (1.2) | 4.4 (2.0) | 2.0 (2.1) |
| PRO-C4 | -32.1 (22.3) | -27.5 (14.0) | 40.1 (17.2) |
| PRO-C6 | 1.0 (0.6) | 1.4 (1.0) | -0.1 (0.9) |
| VICM | 0.2 (1.1) | -3.2 (1.9) | 0.8 (1.2) |

BID, twice daily; CFB, change from baseline; ECM, extracellular matrix; QD, once daily; SEM, standard error of the mean. ECM-neoepitope biomarker abbreviations are defined in Table 1.

**Table S3**. B-H–adjusted *P* values of LRT for treatment*timepoint interactive term significance, and model summaries for all linear mixed models fitted^a,b^

| Biomarker | LRT raw *P* value | LRT B-H–adjusted *P* value | | | | | | | | | | | | | | | | | | | | | |
| --- | --- | --- | --- | --- | --- | --- | --- | --- | --- | --- | --- | --- | --- | --- | --- | --- | --- | --- | --- | --- | --- | --- | --- |
| C1M | **< 0.001** | **< 0.001** | | | | | | | | | | | | | | | | | | | | | |
| C3A | **< 0.001** | **0.002** | | | | | | | | | | | | | | | | | | | | | |
| C3M | **< 0.001** | **< 0.001** | | | | | | | | | | | | | | | | | | | | | |
| C4M2 | **< 0.001** | **< 0.001** | | | | | | | | | | | | | | | | | | | | | |
| C6M | **< 0.001** | **< 0.001** | | | | | | | | | | | | | | | | | | | | | |
| PRO-C3 | **0.001** | **0.002** | | | | | | | | | | | | | | | | | | | | | |
| PRO-C4 | **< 0.001** | **< 0.001** | | | | | | | | | | | | | | | | | | | | | |
| PRO-C6 | **< 0.001** | **< 0.001** | | | | | | | | | | | | | | | | | | | | | |
| VICM | **< 0.001** | **0.002** | | | | | | | | | | | | | | | | | | | | | |
| Model Information | | | | | | | | | | | | | | | | | | | | | | | |
| C1M | Linear mixed model fit by REML ['lmerMod'] | | | | | | | | | | | | | | | | | | | | | | |
|  | Formula: | C1M ~ (1 \| SUBJID) + AGE + SEX + time_c + time_c * TRTP | | | | | | | | | | | | | | | | | | | | | |
|  | Data: | biomarkers_wide | | | | | | | | | | | | | | | | | | | | | |
|  | REML criterion at convergence: | 729.6 | | | | | | | | | | | | | | | | | | | | | |
|  | Scaled residuals: | Min | | | | 1Q | | | | | Median | | | 3Q | | | | | | | Max | | |
|  |  | -2.8432 | | | | -0.5065 | | | | | -0.0811 | | | 0.4162 | | | | | | | 3.6085 | | |
|  | Random effects: | Groups | | | | | | Name | | | | | Variance | | | | | | Std.Dev. | | | | |
|  |  | SUBJID | | | | | | (Intercept) | | | | | 0.2369 | | | | | | 0.4867 | | | | |
|  |  | Residual | | | | | |  | | | | | 0.2389 | | | | | | 0.4888 | | | | |
|  |  | Number of obs: 358, groups: SUBJID, 142 | | | | | | | | | | | | | | | | | | | | | |
|  | Fixed effects: |  | | | | | | Estimate | | | | | Std. Error | | | | | | t value | | | | |
|  |  | (Intercept) | | | | | | 4.5297527 | | | | | 0.4770299 | | | | | | 9.496 | | | | |
|  |  | AGE | | | | | | 0.0013315 | | | | | 0.0066081 | | | | | | 0.202 | | | | |
|  |  | SEXM | | | | | | -0.0435407 | | | | | 0.1086526 | | | | | | -0.401 | | | | |
|  |  | time_c | | | | | | 0.0006071 | | | | | 0.0006218 | | | | | | 0.976 | | | | |
|  |  | TRTPBMS-986020 600 mg QD | | | | | | 0.2519643 | | | | | 0.1317315 | | | | | | 1.913 | | | | |
|  |  | TRTPBMS-986020 600 mg BID | | | | | | -0.0312588 | | | | | 0.1292910 | | | | | | -0.242 | | | | |
|  |  | time_c:TRTPBMS-986020 600 mg QD | | | | | | -0.0005452 | | | | | 0.0008631 | | | | | | -0.632 | | | | |
|  |  | time_c:TRTPBMS-986020 600 mg BID | | | | | | -0.0001225 | | | | | 0.0008717 | | | | | | -0.141 | | | | |
|  | Correlation of fixed effects: |  | | (Intr) | | | | AGE | | | SEXM | | time_c | | | TR6mod | | | TR6mtd | | | | t_6mod |
|  |  | AGE | | -0.967 | | | |  | | |  | |  | | |  | | |  | | | |  |
|  |  | SEXM | | -0.194 | | | | 0.033 | | |  | |  | | |  | | |  | | | |  |
|  |  | time_c | | -0.097 | | | | 0.018 | | | 0.008 | |  | | |  | | |  | | | |  |
|  |  | TRTPBMS6mod | | -0.193 | | | | 0.055 | | | 0.010 | | 0.284 | | |  | | |  | | | |  |
|  |  | TRTPBMS6mtd | | -0.151 | | | | 0.015 | | | -0.028 | | 0.289 | | | 0.510 | | |  | | | |  |
|  |  | t_:TRTP6mod | | 0.084 | | | | -0.028 | | | -0.001 | | -0.721 | | | -0.407 | | | -0.208 | | | |  |
|  |  | t_:TRTP6mtd | | 0.071 | | | | -0.014 | | | -0.015 | | -0.713 | | | -0.203 | | | -0.383 | | | | 0.514 |
| C3A | Linear mixed model fit by REML ['lmerMod'] | | | | | | | | | | | | | | | | | | | | | | |
|  | Formula: | C3A ~ (1 \| SUBJID) + AGE + SEX + time_c + time_c * TRTP | | | | | | | | | | | | | | | | | | | | | |
|  | Data: | biomarkers_wide | | | | | | | | | | | | | | | | | | | | | |
|  | REML criterion at convergence: | 786.6 | | | | | | | | | | | | | | | | | | | | | |
|  | Scaled residuals: | Min | | | 1Q | | | | | Median | | | | | 3Q | | | | | Max | | | |
|  |  | -4.8855 | | | -0.2586 | | | | | 0.0585 | | | | | 0.3114 | | | | | 4.5587 | | | |
|  | Random effects: | Groups | | | | | Name | | | | | Variance | | | | | | Std.Dev. | | | | | |
|  |  | SUBJID | | | | | (Intercept) | | | | | 0.7094 | | | | | | 0.8423 | | | | | |
|  |  | Residual | | | | |  | | | | | 0.1743 | | | | | | 0.4175 | | | | | |
|  |  | Number of obs: 364, groups: SUBJID, 142 | | | | | | | | | | | | | | | | | | | | | |
|  | Fixed effects: |  | | | | | Estimate | | | | | Std. Error | | | | | | t value | | | | | |
|  |  | (Intercept) | | | | | 5.2589337 | | | | | 0.7171588 | | | | | | 7.333 | | | | | |
|  |  | AGE | | | | | 0.0006420 | | | | | 0.0099849 | | | | | | 0.064 | | | | | |
|  |  | SEXM | | | | | -0.2027260 | | | | | 0.1651908 | | | | | | -1.227 | | | | | |
|  |  | time_c | | | | | 0.0010482 | | | | | 0.0005262 | | | | | | 1.992 | | | | | |
|  |  | TRTPBMS-986020 600 mg QD | | | | | 0.5057265 | | | | | 0.1880999 | | | | | | 2.689 | | | | | |
|  |  | TRTPBMS-986020 600 mg BID | | | | | 0.2000252 | | | | | 0.1863443 | | | | | | 1.073 | | | | | |
|  |  | time_c:TRTPBMS-986020 600 mg QD | | | | | -0.0013964 | | | | | 0.0007370 | | | | | | -1.895 | | | | | |
|  |  | time_c:TRTPBMS-986020 600 mg BID | | | | | -0.0011984 | | | | | 0.0007461 | | | | | | -1.606 | | | | | |
|  | Correlation of fixed effects: |  | (Intr) | | | | AGE | | SEXM | | | time_c | | | | | TR6mod | TR6mtd | | | | t_6mod | |
|  |  | AGE | -0.969 | | | |  | |  | | |  | | | | |  |  | | | |  | |
|  |  | SEXM | -0.193 | | | | 0.032 | |  | | |  | | | | |  |  | | | |  | |
|  |  | time_c | -0.046 | | | | 0.002 | | 0.006 | | |  | | | | |  |  | | | |  | |
|  |  | TRTPBMS6mod | -0.175 | | | | 0.046 | | 0.002 | | | 0.162 | | | | |  |  | | | |  | |
|  |  | TRTPBMS6mtd | -0.133 | | | | 0.008 | | -0.041 | | | 0.163 | | | | | 0.503 |  | | | |  | |
|  |  | t_:TRTP6mod | 0.041 | | | | -0.010 | | -0.004 | | | -0.714 | | | | | -0.236 | -0.116 | | | |  | |
|  |  | t_:TRTP6mtd | 0.033 | | | | -0.002 | | -0.009 | | | -0.705 | | | | | -0.114 | -0.220 | | | | 0.504 | |
| C3M | Linear mixed model fit by REML ['lmerMod'] | | | | | | | | | | | | | | | | | | | | | | |
|  | Formula: | C3M ~ (1 \| SUBJID) + AGE + SEX + time_c + time_c * TRTP | | | | | | | | | | | | | | | | | | | | | |
|  | Data: | biomarkers_wide | | | | | | | | | | | | | | | | | | | | | |
|  | REML criterion at convergence: | 272.2 | | | | | | | | | | | | | | | | | | | | | |
|  | Scaled residuals: | Min | | | | 1Q | | | | | Median | | | 3Q | | | | | | | Max | | |
|  |  | -3.2835 | | | | -0.4522 | | | | | -0.0272 | | | 0.4842 | | | | | | | 2.9989 | | |
|  | Random effects: | Groups | | | | | | Name | | | | | Variance | | | | | | Std.Dev. | | | | |
|  |  | SUBJID | | | | | | (Intercept) | | | | | 0.13497 | | | | | | 0.3674 | | | | |
|  |  | Residual | | | | | |  | | | | | 0.04598 | | | | | | 0.2144 | | | | |
|  |  | Number of obs: 364, groups: SUBJID, 142 | | | | | | | | | | | | | | | | | | | | | |
|  | Fixed effects: |  | | | | | | Estimate | | | | | Std. Error | | | | | | t value | | | | |
|  |  | (Intercept) | | | | | | 4.0604889 | | | | | 0.3186753 | | | | | | 12.742 | | | | |
|  |  | AGE | | | | | | -0.0078034 | | | | | 0.0044370 | | | | | | -1.759 | | | | |
|  |  | SEXM | | | | | | -0.0430225 | | | | | 0.0732785 | | | | | | -0.587 | | | | |
|  |  | time_c | | | | | | -0.0001032 | | | | | 0.0002695 | | | | | | -0.383 | | | | |
|  |  | TRTPBMS-986020 600 mg QD | | | | | | 0.0866460 | | | | | 0.0842891 | | | | | | 1.028 | | | | |
|  |  | TRTPBMS-986020 600 mg BID | | | | | | -0.0161339 | | | | | 0.0834130 | | | | | | -0.193 | | | | |
|  |  | time_c:TRTPBMS-986020 600 mg QD | | | | | | -0.0009352 | | | | | 0.0003775 | | | | | | -2.477 | | | | |
|  |  | time_c:TRTPBMS-986020 600 mg BID | | | | | | -0.0006228 | | | | | 0.0003822 | | | | | | -1.629 | | | | |
|  | Correlation of fixed effects: |  | | (Intr) | | | | AGE | | | SEXM | | time_c | | | TR6mod | | | TR6mtd | | | | t_6mod |
|  |  | AGE | | -0.969 | | | |  | | |  | |  | | |  | | |  | | | |  |
|  |  | SEXM | | -0.191 | | | | 0.031 | | |  | |  | | |  | | |  | | | |  |
|  |  | time_c | | -0.052 | | | | 0.003 | | | 0.002 | |  | | |  | | |  | | | |  |
|  |  | TRTPBMS6mod | | -0.176 | | | | 0.046 | | | 0.001 | | 0.186 | | |  | | |  | | | |  |
|  |  | TRTPBMS6mtd | | -0.134 | | | | 0.007 | | | -0.042 | | 0.187 | | | 0.504 | | |  | | | |  |
|  |  | t_:TRTP6mod | | 0.047 | | | | -0.012 | | | -0.001 | | -0.714 | | | -0.270 | | | -0.134 | | | |  |
|  |  | t_:TRTP6mtd | | 0.038 | | | | -0.003 | | | -0.007 | | -0.705 | | | -0.131 | | | -0.253 | | | | 0.504 |
| C4M2 | Linear mixed model fit by REML ['lmerMod'] | | | | | | | | | | | | | | | | | | | | | | |
|  | Formula: | C4M2 ~ (1 \| SUBJID) + AGE + SEX + time_c + time_c * TRTP | | | | | | | | | | | | | | | | | | | | | |
|  | Data: | biomarkers_wide | | | | | | | | | | | | | | | | | | | | | |
|  | REML criterion at convergence: | 172.3 | | | | | | | | | | | | | | | | | | | | | |
|  | Scaled residuals: | Min | | | | 1Q | | | | | Median | | | 3Q | | | | | | | Max | | |
|  |  | -1.67970 | | | | -0.46837 | | | | | -0.09864 | | | 0.46277 | | | | | | | 2.64592 | | |
|  | Random effects: | Groups | | | | | | Name | | | | | Variance | | | | | | Std.Dev. | | | | |
|  |  | SUBJID | | | | | | (Intercept) | | | | | 0.15419 | | | | | | 0.3927 | | | | |
|  |  | Residual | | | | | |  | | | | | 0.05065 | | | | | | 0.2251 | | | | |
|  |  | Number of obs: 144, groups: SUBJID, 77 | | | | | | | | | | | | | | | | | | | | | |
|  | Fixed effects: |  | | | | | | Estimate | | | | | Std. Error | | | | | | t value | | | | |
|  |  | (Intercept) | | | | | | 4.8739808 | | | | | 0.5028290 | | | | | | 9.693 | | | | |
|  |  | AGE | | | | | | -0.0055805 | | | | | 0.0066598 | | | | | | -0.838 | | | | |
|  |  | SEXM | | | | | | -0.0564206 | | | | | 0.1111657 | | | | | | -0.508 | | | | |
|  |  | time_c | | | | | | 0.0005871 | | | | | 0.0005499 | | | | | | 1.068 | | | | |
|  |  | TRTPBMS-986020 600 mg QD | | | | | | 0.2139329 | | | | | 0.1374737 | | | | | | 1.556 | | | | |
|  |  | TRTPBMS-986020 600 mg BID | | | | | | 0.0787670 | | | | | 0.1379651 | | | | | | 0.571 | | | | |
|  |  | time_c:TRTPBMS-986020 600 mg QD | | | | | | -0.0010701 | | | | | 0.0007322 | | | | | | -1.461 | | | | |
|  |  | time_c:TRTPBMS-986020 600 mg BID | | | | | | -0.0013335 | | | | | 0.0007798 | | | | | | -1.710 | | | | |
|  | Correlation of fixed effects: |  | | (Intr) | | | | AGE | | | SEXM | | time_c | | | TR6mod | | | TR6mtd | | | | t_6mod |
|  |  | AGE | | -0.968 | | | |  | | |  | |  | | |  | | |  | | | |  |
|  |  | SEXM | | -0.350 | | | | 0.207 | | |  | |  | | |  | | |  | | | |  |
|  |  | time_c | | -0.080 | | | | -0.008 | | | 0.013 | |  | | |  | | |  | | | |  |
|  |  | TRTPBMS6mod | | -0.270 | | | | 0.134 | | | 0.003 | | 0.312 | | |  | | |  | | | |  |
|  |  | TRTPBMS6mtd | | -0.203 | | | | 0.058 | | | 0.030 | | 0.312 | | | 0.532 | | |  | | | |  |
|  |  | t_:TRTP6mod | | 0.087 | | | | -0.022 | | | -0.018 | | -0.751 | | | -0.451 | | | -0.236 | | | |  |
|  |  | t_:TRTP6mtd | | 0.080 | | | | -0.019 | | | -0.014 | | -0.705 | | | -0.224 | | | -0.442 | | | | 0.530 |
| C6M | Linear mixed model fit by REML ['lmerMod'] | | | | | | | | | | | | | | | | | | | | | | |
|  | Formula: | C6M ~ (1 \| SUBJID) + AGE + SEX + time_c + time_c * TRTP | | | | | | | | | | | | | | | | | | | | | |
|  | Data: | biomarkers_wide | | | | | | | | | | | | | | | | | | | | | |
|  | REML criterion at convergence: | 590.1 | | | | | | | | | | | | | | | | | | | | | |
|  | Scaled residuals: | Min | | | 1Q | | | | | Median | | | | | 3Q | | | | | Max | | | |
|  |  | -5.1134 | | | -0.4797 | | | | | -0.0249 | | | | | 0.4364 | | | | | 4.0258 | | | |
|  | Random effects: | Groups | | | | | Name | | | | | Variance | | | | | | Std.Dev. | | | | | |
|  |  | SUBJID | | | | | (Intercept) | | | | | 0.2463 | | | | | | 0.4963 | | | | | |
|  |  | Residual | | | | |  | | | | | 0.1305 | | | | | | 0.3613 | | | | | |
|  |  | Number of obs: 362, groups: SUBJID, 142 | | | | | | | | | | | | | | | | | | | | | |
|  | Fixed effects: |  | | | | | Estimate | | | | | Std. Error | | | | | | t value | | | | | |
|  |  | (Intercept) | | | | | 4.6054032 | | | | | 0.4458521 | | | | | | 10.329 | | | | | |
|  |  | AGE | | | | | -0.0056088 | | | | | 0.0062072 | | | | | | -0.904 | | | | | |
|  |  | SEXM | | | | | 0.0302786 | | | | | 0.1023205 | | | | | | 0.296 | | | | | |
|  |  | time_c | | | | | 0.0006138 | | | | | 0.0004588 | | | | | | 1.338 | | | | | |
|  |  | TRTPBMS-986020 600 mg QD | | | | | 0.1943685 | | | | | 0.1197511 | | | | | | 1.623 | | | | | |
|  |  | TRTPBMS-986020 600 mg BID | | | | | 0.0996693 | | | | | 0.1182029 | | | | | | 0.843 | | | | | |
|  |  | time_c:TRTPBMS-986020 600 mg QD | | | | | -0.0012424 | | | | | 0.0006385 | | | | | | -1.946 | | | | | |
|  |  | time_c:TRTPBMS-986020 600 mg BID | | | | | -0.0013040 | | | | | 0.0006461 | | | | | | -2.018 | | | | | |
|  | Correlation of Fixed Effects: |  | (Intr) | | | | AGE | | SEXM | | | time_c | | | | | TR6mod | TR6mtd | | | | t_6mod | |
|  |  | AGE | -0.969 | | | |  | |  | | |  | | | | |  |  | | | |  | |
|  |  | SEXM | -0.188 | | | | 0.028 | |  | | |  | | | | |  |  | | | |  | |
|  |  | time_c | -0.065 | | | | 0.007 | | -0.004 | | |  | | | | |  |  | | | |  | |
|  |  | TRTPBMS6mod | -0.178 | | | | 0.046 | | 0.001 | | | 0.220 | | | | |  |  | | | |  | |
|  |  | TRTPBMS6mtd | -0.135 | | | | 0.007 | | -0.041 | | | 0.222 | | | | | 0.504 |  | | | |  | |
|  |  | t_:TRTP6mod | 0.057 | | | | -0.016 | | 0.003 | | | -0.719 | | | | | -0.321 | -0.160 | | | |  | |
|  |  | t_:TRTP6mtd | 0.048 | | | | -0.005 | | -0.004 | | | -0.710 | | | | | -0.156 | -0.301 | | | | 0.510 | |
| PRO-C3 | Linear mixed model fit by REML ['lmerMod'] | | | | | | | | | | | | | | | | | | | | | | |
|  | Formula: | PRO.C3 ~ (1 \| SUBJID) + AGE + SEX + time_c + time_c * TRTP | | | | | | | | | | | | | | | | | | | | | |
|  | Data: | biomarkers_wide | | | | | | | | | | | | | | | | | | | | | |
|  | REML criterion at convergence: | 687.7 | | | | | | | | | | | | | | | | | | | | | |
|  | Scaled residuals: | Min | | | 1Q | | | | | Median | | | | | 3Q | | | | | Max | | | |
|  |  | -2.78323 | | | -0.46220 | | | | | 0.02871 | | | | | 0.50261 | | | | | 2.80238 | | | |
|  | Random effects: | Groups | | | | | Name | | | | | Variance | | | | | | Std.Dev. | | | | | |
|  |  | SUBJID | | | | | (Intercept) | | | | | 0.2403 | | | | | | 0.4902 | | | | | |
|  |  | Residual | | | | |  | | | | | 0.1998 | | | | | | 0.4470 | | | | | |
|  |  | Number of obs: 359, groups: SUBJID, 142 | | | | | | | | | | | | | | | | | | | | | |
|  | Fixed effects: |  | | | | | Estimate | | | | | Std. Error | | | | | | t value | | | | | |
|  |  | (Intercept) | | | | | 3.6718394 | | | | | 0.4738019 | | | | | | 7.750 | | | | | |
|  |  | AGE | | | | | -0.0034937 | | | | | 0.0065801 | | | | | | -0.531 | | | | | |
|  |  | SEXM | | | | | 0.2593512 | | | | | 0.1055085 | | | | | | 2.458 | | | | | |
|  |  | time_c | | | | | 0.0003651 | | | | | 0.0005655 | | | | | | 0.646 | | | | | |
|  |  | TRTPBMS-986020 600 mg QD | | | | | 0.0210348 | | | | | 0.1277662 | | | | | | 0.165 | | | | | |
|  |  | TRTPBMS-986020 600 mg BID | | | | | 0.2364324 | | | | | 0.1263149 | | | | | | 1.872 | | | | | |
|  |  | time_c:TRTPBMS-986020 600 mg QD | | | | | 0.0007890 | | | | | 0.0007921 | | | | | | 0.996 | | | | | |
|  |  | time_c:TRTPBMS-986020 600 mg BID | | | | | 0.0016029 | | | | | 0.0007958 | | | | | | 2.014 | | | | | |
|  | Correlation of fixed effects: |  | (Intr) | | | | AGE | | SEXM | | | time_c | | | | | TR6mod | TR6mtd | | | | t_6mod | |
|  |  | AGE | -0.969 | | | |  | |  | | |  | | | | |  |  | | | |  | |
|  |  | SEXM | -0.173 | | | | 0.020 | |  | | |  | | | | |  |  | | | |  | |
|  |  | time_c | -0.082 | | | | 0.009 | | -0.007 | | |  | | | | |  |  | | | |  | |
|  |  | TRTPBMS6mod | -0.197 | | | | 0.061 | | 0.000 | | | 0.276 | | | | |  |  | | | |  | |
|  |  | TRTPBMS6mtd | -0.154 | | | | 0.023 | | -0.048 | | | 0.279 | | | | | 0.517 |  | | | |  | |
|  |  | t_:TRTP6mod | 0.070 | | | | -0.019 | | 0.008 | | | -0.714 | | | | | -0.384 | -0.200 | | | |  | |
|  |  | t_:TRTP6mtd | 0.060 | | | | -0.007 | | -0.003 | | | -0.711 | | | | | -0.196 | -0.364 | | | | 0.507 | |
| PRO-C4 | Linear mixed model fit by REML ['lmerMod'] | | | | | | | | | | | | | | | | | | | | | | |
|  | Formula: | PRO-C4 ~ (1 \| SUBJID) + AGE + SEX + time_c + time_c * TRTP | | | | | | | | | | | | | | | | | | | | | |
|  | Data: | biomarkers_wide | | | | | | | | | | | | | | | | | | | | | |
|  | REML criterion at convergence: | 451.9 | | | | | | | | | | | | | | | | | | | | | |
|  | Scaled residuals: | Min | | | 1Q | | | | | Median | | | | | 3Q | | | | | Max | | | |
|  |  | -4.7862 | | | -0.4692 | | | | | 0.0212 | | | | | 0.5030 | | | | | 3.5839 | | | |
|  | Random effects: | Groups | | | | | Name | | | | | Variance | | | | | | Std.Dev | | | | | |
|  |  | SUBJID | | | | | (Intercept) | | | | | 0.16215 | | | | | | 0.4027 | | | | | |
|  |  | Residual | | | | |  | | | | | 0.08984 | | | | | | 0.2997 | | | | | |
|  |  | Number of obs: 361, groups: SUBJID, 142 | | | | | | | | | | | | | | | | | | | | | |
|  | Fixed effects: |  | | | | | Estimate | | | | | Std. Error | | | | | | t value | | | | | |
|  |  | (Intercept) | | | | | 7.9854117 | | | | | 0.3718923 | | | | | | 21.472 | | | | | |
|  |  | AGE | | | | | -0.0017183 | | | | | 0.0051671 | | | | | | -0.333 | | | | | |
|  |  | SEXM | | | | | 0.0764057 | | | | | 0.0828757 | | | | | | 0.922 | | | | | |
|  |  | time_c | | | | | 0.0009034 | | | | | 0.0003795 | | | | | | 2.380 | | | | | |
|  |  | TRTPBMS-986020 600 mg QD | | | | | 0.1306779 | | | | | 0.0981733 | | | | | | 1.331 | | | | | |
|  |  | TRTPBMS-986020 600 mg BID | | | | | 0.1078282 | | | | | 0.0973232 | | | | | | 1.108 | | | | | |
|  |  | time_c:TRTPBMS-986020 600 mg QD | | | | | -0.0012633 | | | | | 0.0005285 | | | | | | -2.390 | | | | | |
|  |  | time_c:TRTPBMS-986020 600 mg BID | | | | | -0.0013228 | | | | | 0.0005351 | | | | | | -2.472 | | | | | |
|  | Correlation of fixed effects: |  | (Intr) | | | | AGE | | SEXM | | | time_c | | | | | TR6mod | TR6mtd | | | | t_6mod | |
|  |  | AGE | -0.970 | | | |  | |  | | |  | | | | |  |  | | | |  | |
|  |  | SEXM | -0.175 | | | | 0.022 | |  | | |  | | | | |  |  | | | |  | |
|  |  | time_c | -0.067 | | | | 0.006 | | -0.004 | | |  | | | | |  |  | | | |  | |
|  |  | TRTPBMS6mod | -0.194 | | | | 0.061 | | 0.005 | | | 0.238 | | | | |  |  | | | |  | |
|  |  | TRTPBMS6mtd | -0.151 | | | | 0.024 | | -0.047 | | | 0.240 | | | | | 0.515 |  | | | |  | |
|  |  | t_:TRTP6mod | 0.060 | | | | -0.015 | | 0.000 | | | -0.718 | | | | | -0.333 | -0.172 | | | |  | |
|  |  | t_:TRTP6mtd | 0.049 | | | | -0.004 | | -0.004 | | | -0.709 | | | | | -0.169 | -0.314 | | | | 0.509 | |
| PRO-C6 | Linear mixed model fit by REML ['lmerMod'] | | | | | | | | | | | | | | | | | | | | | | |
|  | Formula: | PRO.C6 ~ (1 \| SUBJID) + AGE + SEX + time_c + time_c * TRTP | | | | | | | | | | | | | | | | | | | | | |
|  | Data: | biomarkers_wide | | | | | | | | | | | | | | | | | | | | | |
|  | REML criterion at convergence: | 557.7 | | | | | | | | | | | | | | | | | | | | | |
|  | Scaled residuals: | Min | | | 1Q | | | | | Median | | | | | 3Q | | | | | Max | | | |
|  |  | -2.9655 | | | -0.5509 | | | | | -0.0162 | | | | | 0.5162 | | | | | 3.3362 | | | |
|  | Random effects: | Groups | | | | | Name | | | | | Variance | | | | | | Std.Dev. | | | | | |
|  |  | SUBJID | | | | | (Intercept) | | | | | 0.2096 | | | | | | 0.4579 | | | | | |
|  |  | Residual | | | | |  | | | | | 0.1066 | | | | | | 0.3265 | | | | | |
|  |  | Number of obs: 397, groups: SUBJID, 143 | | | | | | | | | | | | | | | | | | | | | |
|  | Fixed effects: |  | | | | | Estimate | | | | | Std. Error | | | | | | t value | | | | | |
|  |  | (Intercept) | | | | | 2.7577052 | | | | | 0.4061828 | | | | | | 6.789 | | | | | |
|  |  | AGE | | | | | 0.0101741 | | | | | 0.0056471 | | | | | | 1.802 | | | | | |
|  |  | SEXM | | | | | -0.0644050 | | | | | 0.0927152 | | | | | | -0.695 | | | | | |
|  |  | time_c | | | | | 0.0006017 | | | | | 0.0003760 | | | | | | 1.600 | | | | | |
|  |  | TRTPBMS-986020 600 mg QD | | | | | -0.0967670 | | | | | 0.1077344 | | | | | | -0.898 | | | | | |
|  |  | TRTPBMS-986020 600 mg BID | | | | | 0.1072914 | | | | | 0.1076507 | | | | | | 0.997 | | | | | |
|  |  | time_c:TRTPBMS-986020 600 mg QD | | | | | 0.0002689 | | | | | 0.0005244 | | | | | | 0.513 | | | | | |
|  |  | time_c:TRTPBMS-986020 600 mg BID | | | | | 0.0002836 | | | | | 0.0005303 | | | | | | 0.535 | | | | | |
|  | Correlation of fixed effects: |  | (Intr) | | | | AGE | | SEXM | | | time_c | | | | | TR6mod | TR6mtd | | | | t_6mod | |
|  |  | AGE | -0.969 | | | |  | |  | | |  | | | | |  |  | | | |  | |
|  |  | SEXM | -0.193 | | | | 0.035 | |  | | |  | | | | |  |  | | | |  | |
|  |  | time_c | -0.055 | | | | -0.002 | | -0.006 | | |  | | | | |  |  | | | |  | |
|  |  | TRTPBMS6mod | -0.179 | | | | 0.045 | | 0.009 | | | 0.215 | | | | |  |  | | | |  | |
|  |  | TRTPBMS6mtd | -0.137 | | | | 0.010 | | -0.042 | | | 0.215 | | | | | 0.505 |  | | | |  | |
|  |  | t_:TRTP6mod | 0.034 | | | | 0.006 | | 0.005 | | | -0.717 | | | | | -0.301 | -0.154 | | | |  | |
|  |  | t_:TRTP6mtd | 0.042 | | | | -0.002 | | 0.000 | | | -0.709 | | | | | -0.152 | -0.299 | | | | 0.508 | |
| VICM | Linear mixed model fit by REML ['lmerMod'] | | | | | | | | | | | | | | | | | | | | | | |
|  | Formula: | VICM ~ (1 \| SUBJID) + AGE + SEX + time_c + time_c * TRTP | | | | | | | | | | | | | | | | | | | | | |
|  | Data: | biomarkers_wide | | | | | | | | | | | | | | | | | | | | | |
|  | REML criterion at convergence: | 1251.1 | | | | | | | | | | | | | | | | | | | | | |
|  | Scaled residuals: | Min | | | | 1Q | | | | | Median | | | 3Q | | | | | | | Max | | |
|  |  | -2.92774 | | | | -0.60484 | | | | | 0.00091 | | | 0.52383 | | | | | | | 2.61688 | | |
|  | Random effects: | Groups | | | | | | Name | | | | | Variance | | | | | | Std.Dev. | | | | |
|  |  | SUBJID | | | | | | (Intercept) | | | | | 1.1280 | | | | | | 1.0621 | | | | |
|  |  | Residual | | | | | |  | | | | | 0.9563 | | | | | | 0.9779 | | | | |
|  |  | Number of obs: 364, groups: SUBJID, 142 | | | | | | | | | | | | | | | | | | | | | |
|  | Fixed effects: |  | | | | | | Estimate | | | | | Std. Error | | | | | | t value | | | | |
|  |  | (Intercept) | | | | | | 0.2556789 | | | | | 1.0057657 | | | | | | 0.254 | | | | |
|  |  | AGE | | | | | | 0.0142485 | | | | | 0.0139977 | | | | | | 1.018 | | | | |
|  |  | SEXM | | | | | | 0.4381826 | | | | | 0.2299275 | | | | | | 1.906 | | | | |
|  |  | time_c | | | | | | 0.0003873 | | | | | 0.0012170 | | | | | | 0.318 | | | | |
|  |  | TRTPBMS-986020 600 mg QD | | | | | | 0.2073970 | | | | | 0.2754111 | | | | | | 0.753 | | | | |
|  |  | TRTPBMS-986020 600 mg BID | | | | | | 0.0063507 | | | | | 0.2714936 | | | | | | 0.023 | | | | |
|  |  | time_c:TRTPBMS-986020 600 mg QD | | | | | | 0.0015857 | | | | | 0.0017041 | | | | | | 0.931 | | | | |
|  |  | time_c:TRTPBMS-986020 600 mg BID | | | | | | -0.0011617 | | | | | 0.0017267 | | | | | | -0.673 | | | | |
|  | Correlation of fixed effects: |  | | (Intr) | | | | AGE | | | SEXM | | time_c | | | TR6mod | | | TR6mtd | | | | t_6mod |
|  |  | AGE | | -0.968 | | | |  | | |  | |  | | |  | | |  | | | |  |
|  |  | SEXM | | -0.186 | | | | 0.027 | | |  | |  | | |  | | |  | | | |  |
|  |  | time_c | | -0.075 | | | | 0.004 | | | 0.002 | |  | | |  | | |  | | | |  |
|  |  | TRTPBMS6mod | | -0.180 | | | | 0.045 | | | 0.001 | | 0.261 | | |  | | |  | | | |  |
|  |  | TRTPBMS6mtd | | -0.138 | | | | 0.006 | | | -0.040 | | 0.264 | | | 0.505 | | |  | | | |  |
|  |  | t_:TRTP6mod | | 0.066 | | | | -0.016 | | | 0.001 | | -0.714 | | | -0.377 | | | -0.189 | | | |  |
|  |  | t_:TRTP6mtd | | 0.055 | | | | -0.003 | | | -0.010 | | -0.705 | | | -0.184 | | | -0.356 | | | | 0.503 |

^a^ *P* < 0.05 values are shown in bold. ^b^ The LRT tests whether we can predict the biomarker level significantly better by including treatment*timepoint as a covariate, versus a null model that ignores treatment*timepoint effects. Model summaries include the terms "time c:TRTPBMS-986020 600 mg QD" and "time_c:TRTPBMS-986020 600 mg BID" that describe the average per day effect of each BMS-986020 dose compared with placebo. B-H, Benjamini-Hochberg; BID, twice daily; LRT, likelihood ratio test; QD, once daily.

**Figure S3.** Pairwise Spearman and Pearson correlation analyses of Week 26 CFB in clinical laboratory liver biomarkers and ECM-neoepitope biomarkers in patients treated with BMS-986020


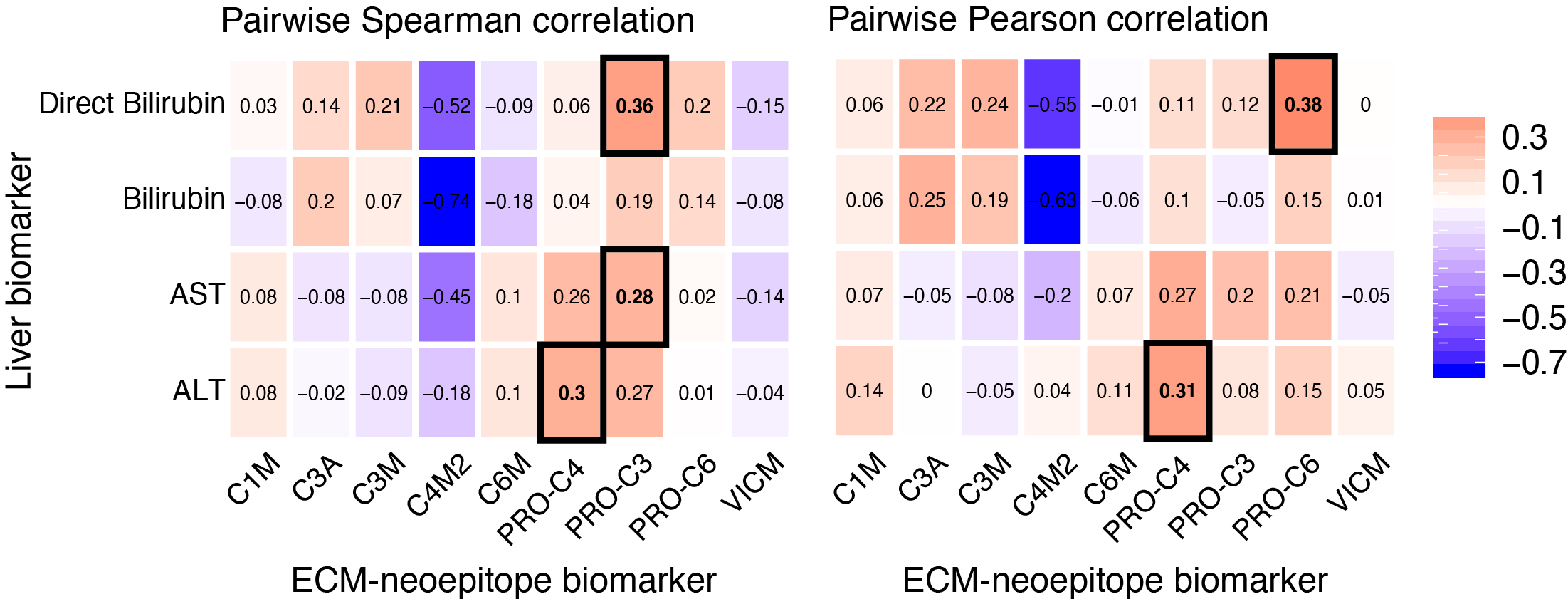


ALT, alanine aminotransferase; AST, aspartate aminotransferase; CFB, change from baseline; ECM, extracellular matrix.

**Table S4.** Kruskal-Wallis one-way analysis of variance and B-H–adjusted *P* values for each group tested^a^

| Stimulant | Day | Biomarker | K-W | Adjusted *P* value |
| --- | --- | --- | --- | --- |
| LPA | Day 4 | a-SMA | NaN | NaN |
| LPA | Day 4 | FBN-C | 0.013 | **0.040** |
| LPA | Day 4 | PRO-C1 | < 0.001 | **0.003** |
| LPA | Day 4 | PRO-C3 | 0.001 | **0.013** |
| LPA | Day 4 | PRO-C6 | < 0.001 | **0.003** |
| LPA | Day 8 | a-SMA | < 0.001 | **0.009** |
| LPA | Day 8 | FBN-C | < 0.001 | **0.009** |
| LPA | Day 8 | PRO-C1 | < 0.001 | **0.004** |
| LPA | Day 8 | PRO-C3 | < 0.001 | **0.003** |
| LPA | Day 8 | PRO-C6 | < 0.001 | **0.004** |
| LPA | Day 12 | a-SMA | < 0.001 | **0.003** |
| LPA | Day 12 | FBN-C | < 0.001 | **0.003** |
| LPA | Day 12 | PRO-C1 | < 0.001 | **0.004** |
| LPA | Day 12 | PRO-C3 | < 0.001 | **0.004** |
| LPA | Day 12 | PRO-C6 | < 0.001 | **0.003** |
| TGF-b1 | Day 4 | a-SMA | 0.149 | 0.298 |
| TGF-b1 | Day 4 | FBN-C | 0.336 | 0.336 |
| TGF-b1 | Day 4 | PRO-C1 | < 0.001 | **0.008** |
| TGF-b1 | Day 4 | PRO-C3 | 0.002 | **0.015** |
| TGF-b1 | Day 4 | PRO-C6 | 0.008 | **0.031** |
| TGF-b1 | Day 8 | a-SMA | < 0.001 | **0.009** |
| TGF-b1 | Day 8 | FBN-C | < 0.001 | **0.006** |
| TGF-b1 | Day 8 | PRO-C1 | < 0.001 | **0.005** |
| TGF-b1 | Day 8 | PRO-C3 | 0.006 | **0.029** |
| TGF-b1 | Day 8 | PRO-C6 | < 0.001 | **0.008** |
| TGF-b1 | Day 12 | a-SMA | 0.001 | **0.013** |
| TGF-b1 | Day 12 | FBN-C | 0.001 | **0.013** |
| TGF-b1 | Day 12 | PRO-C1 | < 0.001 | **0.009** |
| TGF-b1 | Day 12 | PRO-C3 | 0.002 | **0.014** |
| TGF-b1 | Day 12 | PRO-C6 | 0.001 | **0.013** |

^a^ *P* < 0.05 values are shown in bold. B-H, Benjamini-Hochberg; K-W, Kruskal-Wallis; LPA, lysophosphatidic acid; NaN, not a number; TGF-b1, transforming growth factor beta 1.

**Table S5.** Adjusted *P* values from Dunnett's test of untreated and each BMS-986020 dose with vehicle, for all combinations of fibrosis induction, timepoint, and biomarker in a Scar-in-a-Jar model^a,b^

| 5 µM BMS-986020/veh | 0.983 | 1.000 | 0.960 | **< 0.001** | **0.008** | **0.007** | **< 0.001** | **< 0.001** | 0.973 | **0.004** | 0.112 | **0.001** | **< 0.001** | 0.709 | **<0.001** | **0.003** | 0.199 | **0.003** | **< 0.001** | **0.009** | **0.032** | **0.016** | **0.008** | **0.003** | **0.002** | 0.967 | 0.226 |
| --- | --- | --- | --- | --- | --- | --- | --- | --- | --- | --- | --- | --- | --- | --- | --- | --- | --- | --- | --- | --- | --- | --- | --- | --- | --- | --- | --- |
| 1 µM BMS-986020/veh | 0.051 | **< 0.001** | 0.303 | **< 0.001** | **< 0.001** | **0.010** | **< 0.001** | **< 0.001** | **< 0.001** | **< 0.001** | **< 0.001** | **< 0.001** | **< 0.001** | **< 0.001** | **0.047** | 0.140 | **0.011** | **0.008** | **< 0.001** | 0.959 | 0.557 | **< 0.001** | **0.005** | 0.157 | **0.004** | 0.169 | **0.007** |
| 0.5 µM BMS-986020/veh | **0.035** | **< 0.001** | 0.286 | **< 0.001** | **< 0.001** | **< 0.001** | **< 0.001** | **< 0.001** | **< 0.001** | **< 0.001** | **< 0.001** | **< 0.001** | **< 0.001** | **< 0.001** | 0.183 | 0.613 | 0.120 | 0.059 | **< 0.001** | 0.148 | 0.892 | **< 0.001** | 0.696 | 0.068 | **0.011** | **0.030** | **0.013** |
| 0.1 µM BMS-986020/veh | 0.563 | **< 0.001** | 0.319 | **< 0.001** | **< 0.001** | 0.055 | **< 0.001** | **< 0.001** | **< 0.001** | **< 0.001** | **< 0.001** | **< 0.001** | **< 0.001** | **< 0.001** | **0.006** | **0.027** | **0.013** | **0.003** | **< 0.001** | **< 0.001** | 0.135 | **< 0.001** | 0.895 | **0.023** | **0.028** | **0.043** | **0.033** |
| 0.05 µM BMS-986020/veh | 0.772 | **< 0.001** | 0.396 | **< 0.001** | 0.059 | 0.131 | **< 0.001** | **< 0.001** | **< 0.001** | **< 0.001** | **< 0.001** | 0.367 | **< 0.001** | **< 0.001** | **< 0.001** | 0.127 | 0.112 | **0.011** | **< 0.001** | 0.107 | 0.131 | **0.018** | 0.997 | **0.004** | 0.597 | **0.042** | **0.004** |
| 0.01 µM BMS-986020/veh | 0.907 | 0.496 | 0.717 | 0.725 | 0.902 | 0.681 | 0.986 | 0.998 | **< 0.001** | 0.588 | **0.015** | 0.951 | **< 0.001** | **0.015** | **0.003** | **0.021** | **0.045** | 0.103 | **< 0.001** | **0.004** | 0.458 | **0.009** | 0.997 | 0.058 | 0.879 | 0.055 | **0.009** |
| Untreated/veh | 0.983 | 1.000 | 0.960 | **< 0.001** | **0.008** | **0.007** | **< 0.001** | **< 0.001** | 0.973 | **0.004** | 0.112 | **0.001** | **< 0.001** | 0.709 | **< 0.001** | **0.003** | 0.199 | **0.003** | **< 0.001** | **0.009** | **0.032** | **0.016** | **0.008** | **0.003** | **0.002** | 0.967 | 0.226 |
| Biomarker | FBN-C | PRO-C1 | PRO-C3 | PRO-C6 | a-SMA | FBN-C | PRO-C1 | PRO-C3 | PRO-C6 | a-SMA | FBN-C | PRO-C1 | PRO-C3 | PRO-C6 | PRO-C1 | PRO-C3 | PRO-C6 | a-SMA | FBN-C | PRO-C1 | PRO-C3 | PRO-C6 | a-SMA | FBN-C | PRO-C1 | PRO-C3 | PRO-C6 |
| Day | Day 4 | Day 4 | Day 4 | Day 4 | Day 8 | Day 8 | Day 8 | Day 8 | Day 8 | Day 12 | Day 12 | Day 12 | Day 12 | Day 12 | Day 4 | Day 4 | Day 4 | Day 8 | Day 8 | Day 8 | Day 8 | Day 8 | Day 12 | Day 12 | Day 12 | Day 12 | Day 12 |
| Stimulant | LPA | LPA | LPA | LPA | LPA | LPA | LPA | LPA | LPA | LPA | LPA | LPA | LPA | LPA | TGF-b1 | TGF-b1 | TGF-b1 | TGF-b1 | TGF-b1 | TGF-b1 | TGF-b1 | TGF-b1 | TGF-b1 | TGF-b1 | TGF-b1 | TGF-b1 | TGF-b1 |

^a^ *P* < 0.05 values are shown in bold. ^b^ The conditions with a significant Kruskal-Wallis test (e-Table 2) are presented.
LPA, lysophosphatidic acid; TGF-b1, transforming growth factor beta 1; Veh, vehicle.

**Figure S4.** Alamar Blue-measured metabolic activity of LPA- and TGF-β1–stimulated fibroblasts at Day 0 and Day 12, stratified by treatment


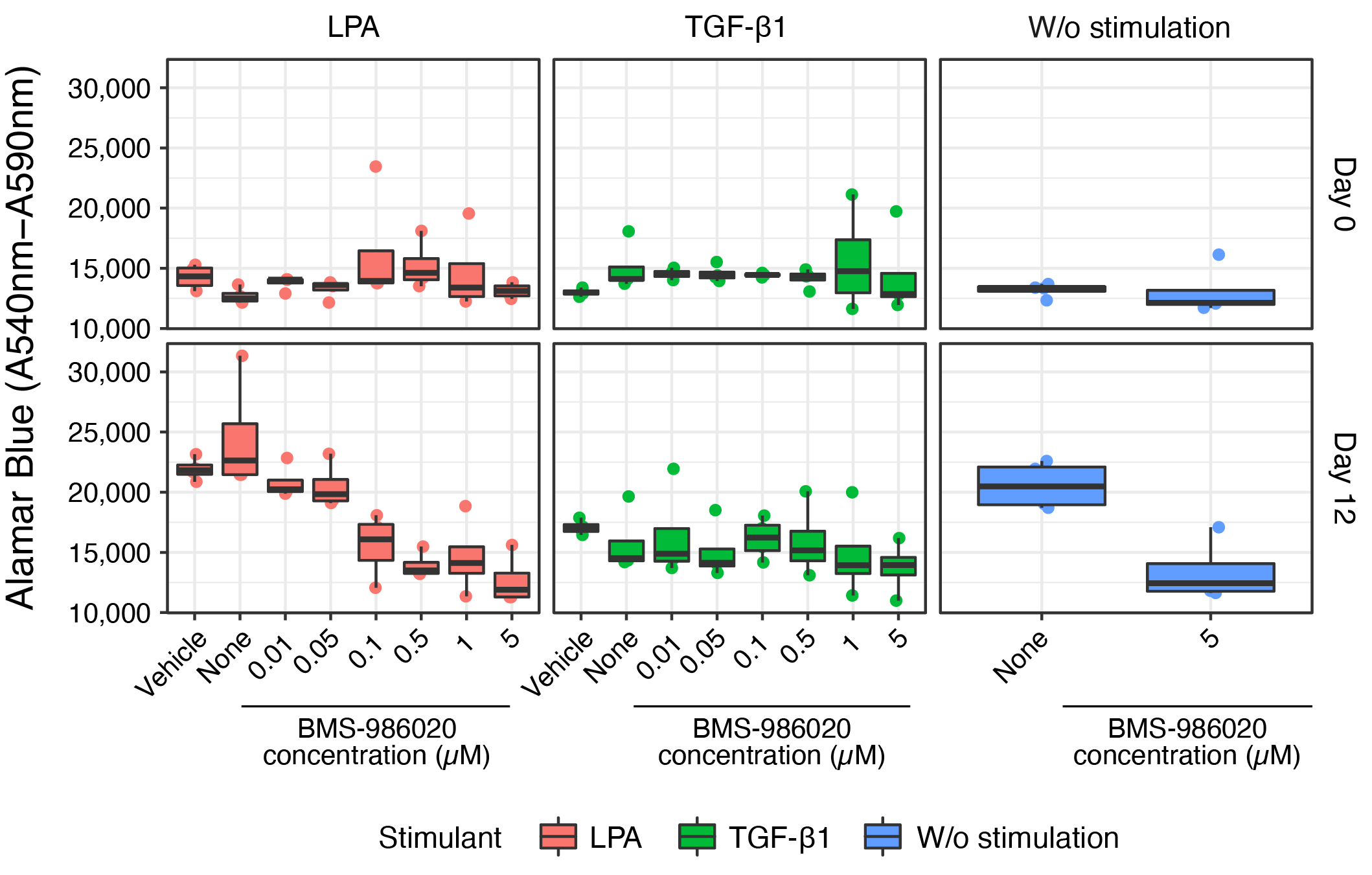


LPA, lysophosphatidic acid; TGF-β1, transforming growth factor-beta 1.

**Table S6.** Alamar Blue Kruskal-Wallis test raw and adjusted *P* values (A) and likelihood ratio test *P* values testing for significant improvement to model fit when including BMS-986020 treatment status in unstimulated group (B)

| A. | Day | Stimulant | *P* value^a^ | Adjusted *P* value^a^ |
| --- | --- | --- | --- | --- |
|  | Day 0 | LPA | 0.082 | 0.245 |
|  | Day 0 | TGF-b1 | 0.243 | 0.486 |
|  | Day 0 | w/o stimulation | 0.248 | 0.486 |
|  | Day 12 | LPA | **< 0.001** | **0.002** |
|  | Day 12 | TGF-b1 | 0.414 | 0.414 |
|  | Day 12 | w/o stimulation | **0.021** | **0.042** |
| B. | Biomarker | *P* value, likelihood ratio test^a^ | B-H–adjusted *P* value^a^ |  |
|  | a-SMA | **0.001** | **0.001** |  |
|  | FBN-C | 0.166 | 0.166 |  |
|  | PRO-C1 | **0.025** | **0.025** |  |
|  | PRO-C3 | **0.029** | **0.029** |  |
|  | PRO-C6 | **< 0.001** | **< 0.001** |  |

^a^ *P* < 0.05 values are shown in bold. B-H, Benjamini-Hochberg; LPA, lysophosphatidic acid; TGF-b1, transforming growth factor beta 1.

**Figure S5:** Fold change between untreated, vehicle-treated, and BMS-986020–treated levels of each ECM-neoepitope biomarker


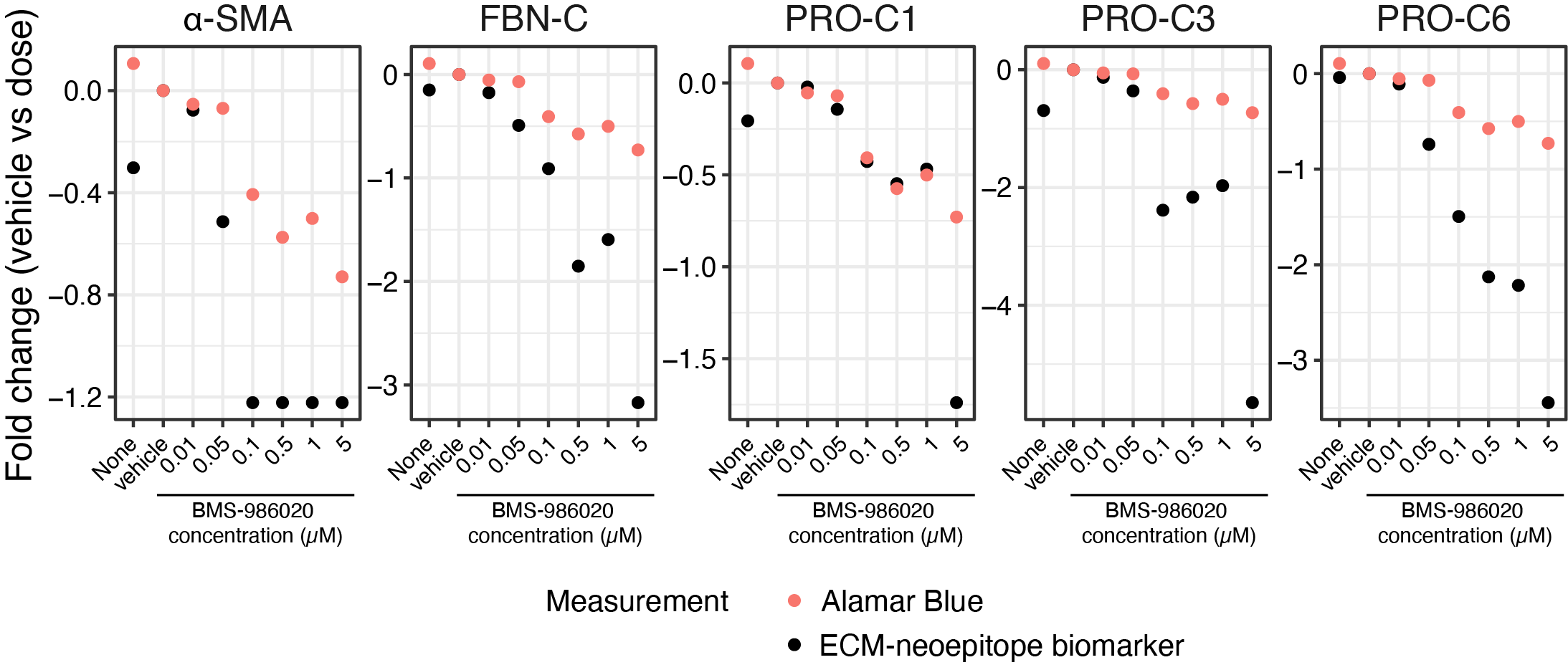


ECM, extracellular matrix.

**Figure S6:** Linear regression of change in ECM-neoepitope biomarker levels over time by treatment in unstimulated Scar-in-a-Jar primary fibroblasts


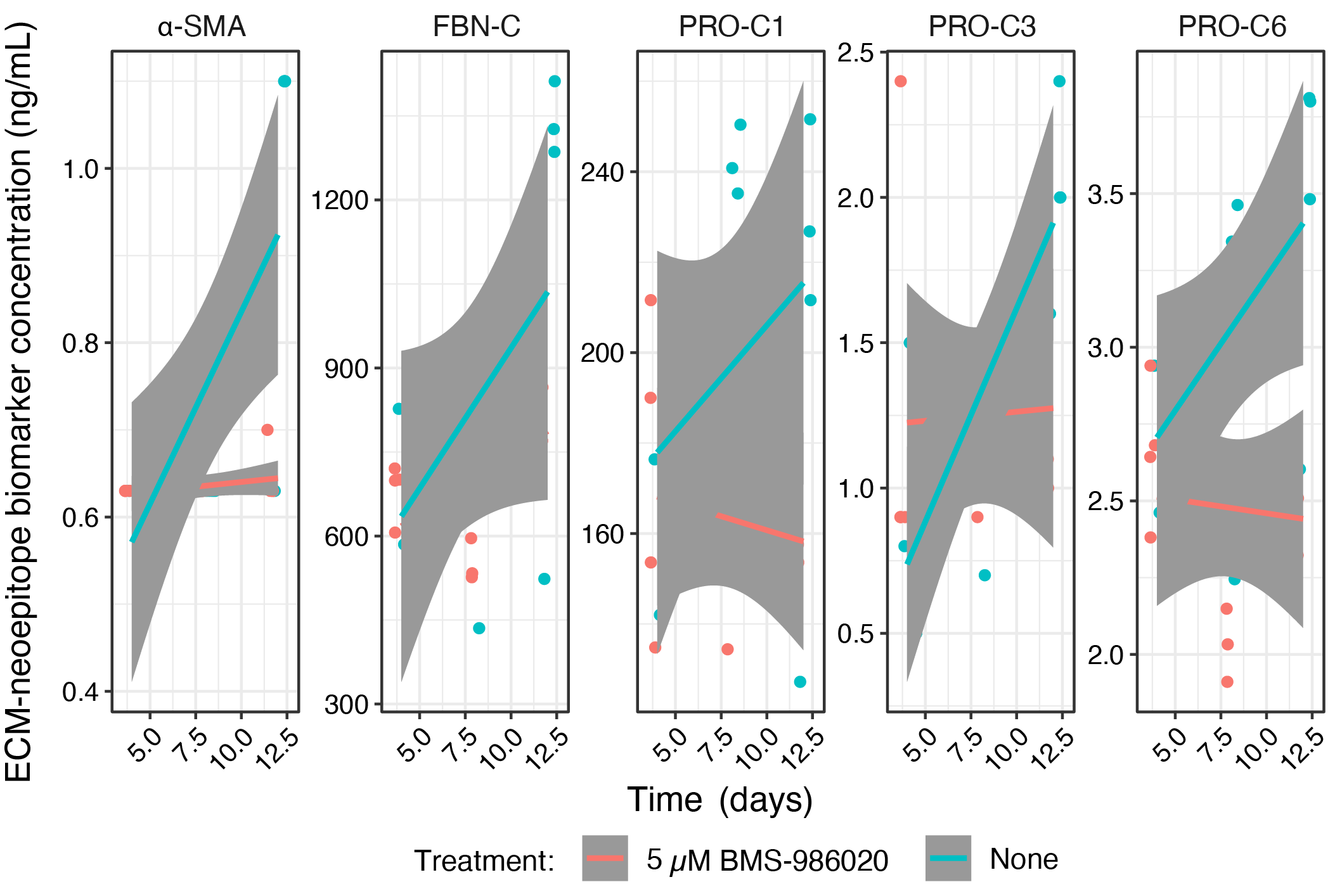


Comparison of no treatment to 5 μM BMS-986020 was done using a likelihood ratio test between linear models with and without the treatment information. A significantly better fit with BMS-986020 for all but FBN-C was observed. ECM, extracellular matrix.
